# A modeling method for thermal steady-state simulation of the four-layer printed circuit board

**DOI:** 10.1371/journal.pone.0310237

**Published:** 2024-09-18

**Authors:** Yabin Zhang, Lin Chen

**Affiliations:** School of Electrical Engineering and Electronic Information, Xihua University, Chengdu, Sichuan, P.R. China; inSync Mirror LLC, UNITED STATES OF AMERICA

## Abstract

Thermal simulation of a Printed Circuit Board (PCB) can help identify potential overheating risks in the circuit. The proposed modeling method combines analytical temperature solutions and numerical approximations. Only Fourier-series analytical solutions related to the prepreg-layer surfaces need to be calculated, rather than the entire structure. Heat transfer through the lateral sides of a PCB is approximately considered as part of the compensated heat flux of the insulating-layer surface boundaries. Heat diffusion within or between metal layers is numerically approximated using the finite volume method. The core layer is treated as “thermally-thick”. Temperature-dependent boundary conditions are considered through iterations. A test solver was developed based on the method. The modeling accuracy was validated by comparison with COMSOL Multiphysics for a four-layer structure with a moderate degree of discretization. Additionally, a PCB for generating DC 3.3V was designed, tested, and modeled, with the modeling results confirmed by the thermal images. The electro-thermal analysis of the distribution of electric potential and Joule heating in traces and vias was integrated into the PCB model. The layout maps of the PCB were further adjusted to reduce Joule heating in the output circuit, and the improvement on reducing the IR drop and hotspot temperature was examined.

## 1. Introduction

Thermal simulation of Printed Circuit Boards (PCBs) during the design stage can help optimize layouts [1–7] and identify potential risks of thermal runaway, ultimately improving circuit reliability [8]. Localized overstress may also be found if combined with mechanical analysis [9]. One way to perform such simulations is through mathematical modeling based on analyzing the discrete thermal resistances [2,6,10] or discrete effective thermal conductivities [11–13] of a PCB, which usually enables the construction of a thermal resistance network for the PCB. A simplified four-layer PCB model was developed to predict SiC MOSFET temperatures for cooling design in an integrated motor drive [10]. Only the small PCBs with thermal vias attached to MOSFETS underwent thermal resistance discretization, while the main board was simplified into a single vertical thermal resistance for calculating the equivalent heat transfer coefficient (HTC) [10]. This approach facilitated temperature calculations under forced air cooling. However, if lateral thermal conduction in metal layers and other components was considered, the accuracy of the predicted temperatures may be further improved. Considering the trace direction of each discrete region, a continuity check approach was also developed [11]. The trace widths at four sub-area boundaries were checked and compared to verify the trace continuity [11]. But the typical pattern with two separate bending sub-traces of the same width may be treated as two intersecting traces, leading to an over-estimated effective thermal conductivity. A neural network algorithm has also been employed to predict the effective thermal conductivity map of semiconductor package substrates [12], but the accuracy may depend on the quality of the learning data and the level of discreteness.

Although the computational efficiency of these methods can be higher compared to the detailed modeling using FEM-based method, the accuracy may be compromised because each trace layout pattern may not always be evenly distributed or fully considered in each discrete region. Moreover, in these models, heat transfer through the lateral sides of a PCB or substrate is usually not considered, thus this ignorance is also a potential factor that influences modeling accuracy.

Certainly, several commercial software, such as FloTHERM, Ansys Icepak, COMSOL Multiphysics and Celsius Thermal Solver, can also be utilized to simulate the thermal behaviour of PCBs [1–7,13–15]. FloTHERM calculates the regional effective thermal conductivities of a PCB approximately by analyzing the regional cover rates of metal-foil layers [13]. But the accuracy of this strategy may heavily rely on the discreteness of the structure. Other software is mainly based on the finite element method (FEM). Results of FEM-based software are usually considered to be agreeable. However, they usually require the complete discretization of the structure, which may reduce computational efficiency [15].

**Fig 1:**
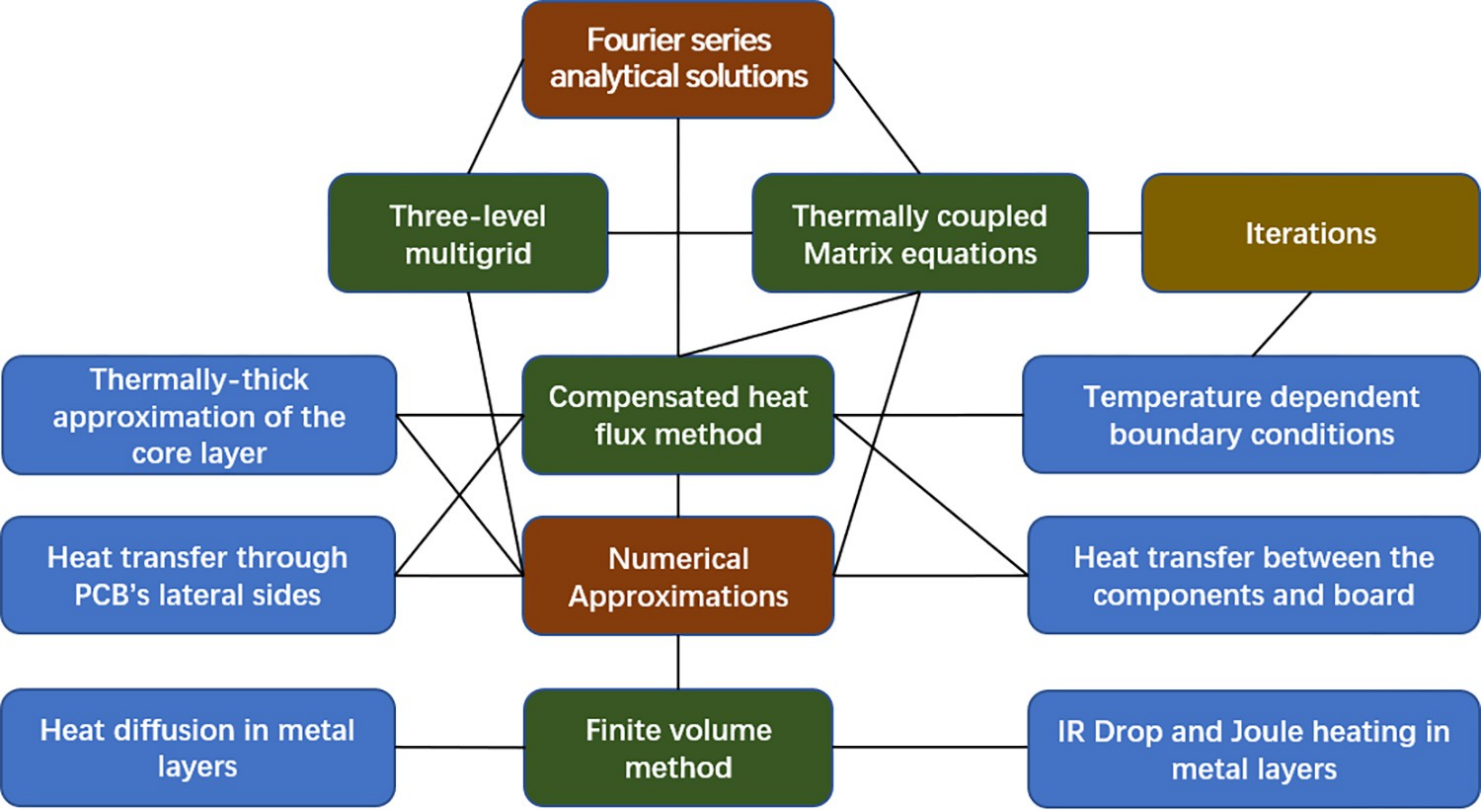
Functions and approaches included in the modeling method.

As shown in Fig 1, the proposed modeling method is based on combining the analytical solution of temperature with numerical approximations. Each line indicates a direct relationship between two connected blocks. The brown blocks represent the two main categories of approaches on which the modeling method is based. The green blocks represent the conventional methods involved. The blue blocks represent the calculations specifically related to numerical discretization approximations. The coffee block “Iterations” specifically indicates that when temperature-related thermal boundary conditions are present, the coupled thermal equations will undergo iterative calculations. The benefit of identifying analytical solutions is avoiding complete discretization of the PCB structure, but only the metal layers and their attached insulating surfaces [16,17]. The analytical solutions take the form of Fourier series. Other similar analytical approaches have been utilized to establish thermal models of power devices [18–21], an LED module [22,23] and a heat spreader [24] as well as a cooling mechanism for microprocessor chips [25].

Since there are both metal layers and insulating layers in a PCB, the inhomogeneous structure prevents the use of analytical solutions alone for thermal analysis calculations and necessitates the inclusion of numerical approximations. Numerical approximations involve the compensated heat flux method used to correct assumed unrealistic average HTCs, the finite volume method for approximating heat diffusion and electric potential distribution within metal layers, the multigrid method for enhancing operational efficiency, considering discrete thermal resistance between ICs or components and the board, the “thermally-thick” approximation of the core layer, and iterations to address temperature-dependent boundary conditions and calculate Joule heating in the circuit. Most of the approaches are discussed in Section 2, along with the introduction of a test solver developed according to the method. A four-layer structure was modeled using both the test solver and COMSOL Multiphysics to assess the method’s feasibility and accuracy. The corresponding comparison is presented in Section 3. A PCB for generating DC 3.3V was also modeled and tested, and the PCB layouts were further improved. The corresponding model and experiment are provided in Section 4.

## 2. Fundamentals of the modeling method

**Fig 2:**
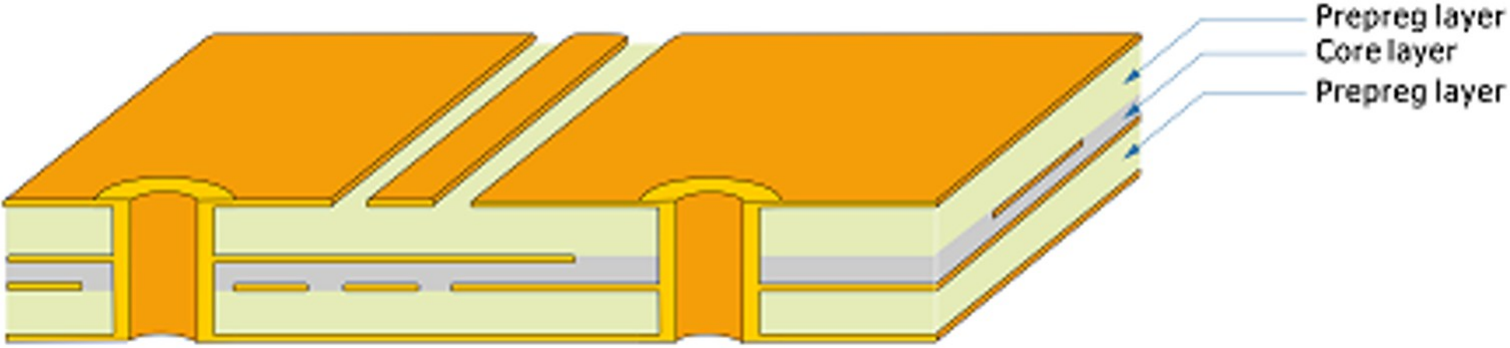
Cross-section schematic of the four-layer PCB structure.

As depicted in Fig 2, the four-layer stack consists of two prepreg layers, and one core layer, with each prepreg layer attached to two metal layers. Thus, the structure can also be viewed as two “double-sided PCB” units bonded together through the core. In addition, the temperature distribution of the lateral sides of each thin insulating layer is approximated as the average of the boundary temperature distributions of both its upper and lower surfaces. In this way, heat transfer through the lateral sides of a PCB can thus be considered as one part of the surface compensated-heat-flux.

Since the thermal conductivity of the FR-4 core is significantly lower than that of the attached metal layer, the core layer is assumed as a “thermally-thick” layer. Thus, only vertical heat conduction through the core is considered. A similar approximation method can also be found in COMSOL Multiphysics for modeling a poor thermal conductor with much lower thermal conductivity compared to the adjacent geometry. In this way, the two bonded “double-sided PCB” can be modeled together.

### 2.1 Analytical solutions of the prepreg layer

To model each double-sided structure attached to the core, the analytical solution of the prepreg layer is represented using Fourier series, and heat diffusion in the two metal-foil layers is numerically approximated. These representations are then converted into matrix equations.

**Fig 3:**
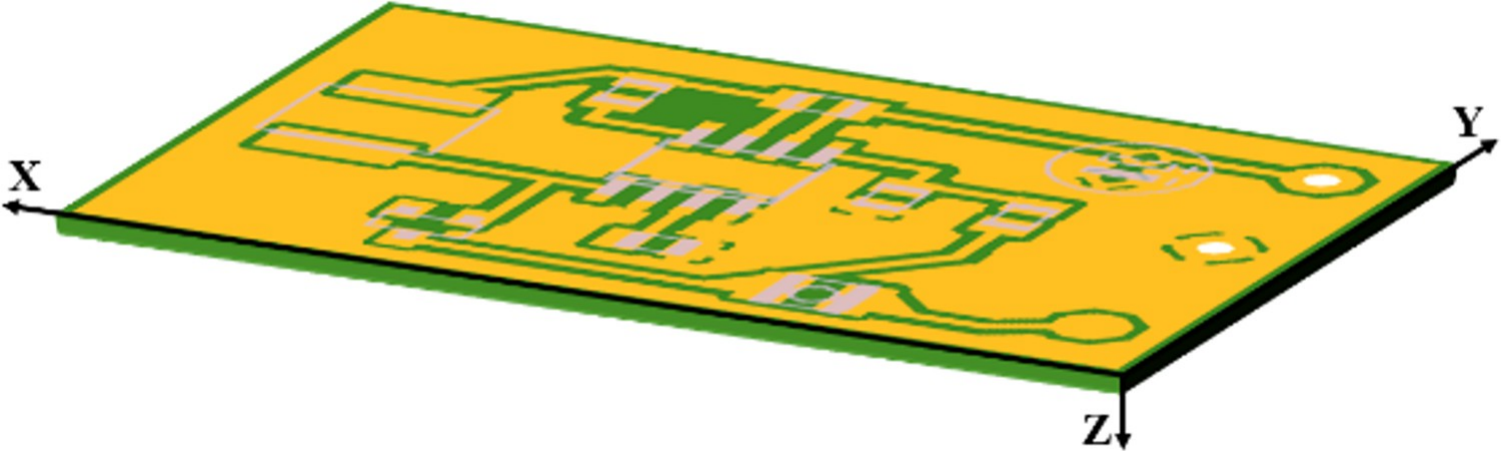
Schematic of a PCB under Cartesian coordinates.


Despite the non-uniformity of the prepreg layer caused by the metal vias, their impact on the lateral conduction of heat can be ignored due to the presence of insulation material around them [16]. Additionally, vias’ contribution to heat transfer between two connected metal layers can be accounted for using the approximation of discrete vertical thermal resistance, which will be explained in Section 2.3. Therefore, the governing equation for steady-state heat diffusion in the prepreg layer under Cartesian coordinates (illustrated in Fig 3) can be approximated by a Laplace’s equation:

$$\nabla^{2}T=\frac{\partial^{2}T}{\partial x^{2}}+\frac{\partial^{2}T}{\partial y^{2}}+\frac{\partial^{2}T}{\partial z^{2}}=0,$$ (1)

where *T* denotes the increase in temperature. The corresponding thermal boundary conditions can be assumed firstly as follows:

$$\left\{ \begin{array}{l} -k_{i}\frac{\partial T}{\partial x}=0\{x=0,x=L_{x}\},\;-k_{i}\frac{\partial T}{\partial y}=0\{y=0,y=L_{y}\},\\ -k_{i}\frac{\partial T}{\partial z}=q_{iu}(x,y)-h_{u}T\{z=0\},\;-k_{i}\frac{\partial T}{\partial z}=q_{id}(x,y)+h_{d}T\{z=L_{in}\}, \end{array}\right.$$ (2)

where *L*_*x*_ and *L*_*y*_ denote the dimensions along *X-* and *Y-*axis, respectively; *L*_*in*_ denotes the thickness; *k*_*i*_ denotes the thermal conductivity of the prepreg layer; *q*_*iu*_*(x*,*y)* denotes the vertical heat flux at the upper surface (*z = 0*) and *q*_*id*_*(x*,*y)* denotes that at the lower surface (*z = L*_*in*_); *h*_*u*_ and *h*_*d*_ denote the initial assumed average HTCs of the upper and lower surfaces, respectively. In the four-layer PCB, obviously, *h*_*d*_ of the top prepreg layer is zero, and *h*_*u*_ of the bottom prepreg layer is zero. Consideration of heat transfer through the four lateral sides (*x = 0*, *x = L*_*x*_, *y = 0*, *and y = L*_*y*_) will be explained in the next section.

By applying the superposition theorem of thermal effect, Eq (1) can be divided into two sub-equations:

$$\left\{ \begin{array}{l} T=\theta +\eta ,\\ \nabla^{2}\theta =0,\\ -k_{i}\frac{\partial \theta }{\partial x}=0\{x=0,x=L_{x}\},\;-k_{i}\frac{\partial \theta }{\partial y}=0\{y=0,y=L_{y}\},\\ -k_{i}\frac{\partial \theta }{\partial z}=q_{iu}(x,y)-h_{u}\theta \{z=0\},\;-k_{i}\frac{\partial \theta }{\partial z}=h_{d}\theta \{z=L_{in}\},\\ \nabla^{2}\eta =0,\\ -k_{i}\frac{\partial \eta }{\partial x}=0\{x=0,x=L_{x}\},\;-k_{i}\frac{\partial \eta }{\partial y}=0\{y=0,y=L_{y}\},\\ -k_{i}\frac{\partial \eta }{\partial z}=-h_{u}\eta \{z=0\},\;-k_{i}\frac{\partial \eta }{\partial z}=q_{id}(x,y)+h_{d}\eta \{z=L_{in}\}, \end{array}\right.$$ (3)

where *θ* and *η* are sub-variables of *T* related to *q*_*iu*_*(x*,*y)* and *q*_*id*_*(x*,*y)*, respectively.

To the extent that each group of differential equations is homogenous, their analytical solutions can be expressed as the product of three sub-solutions that are related to only one variable each. For example, the general solution of *θ* can be expressed as follows:

$$\theta =\sum_{n=0}^{\infty }\sum_{m=0}^{\infty }X_{\theta \_n}(x)Y_{\theta \_m}(y)Z_{\theta \_n,m}(z).$$ (4)

Next, by substituting each group of sub-solutions back into the heat diffusion equation for *θ* and dividing the corresponding group, the equation is converted to the following form:

$$\frac{\partial^{2}X_{\theta \_n}(x)}{\partial x^{2}X_{\theta \_n}(x)}+\frac{\partial^{2}Y_{\theta \_m}(y)}{\partial x^{2}Y_{\theta \_m}(y)}+\frac{\partial^{2}Z_{\theta \_n,m}(z)}{\partial x^{2}Z_{\theta \_n,m}(z)}=0.$$ (5)

Since each term of (5) only contains only one independent variable, they must remain constant when any group of (x,y,z) is substituted into the equation:

$$\left\{ \begin{array}{l} \frac{\partial^{2}X_{\theta \_n}(x)}{\partial x^{2}X_{\theta \_n}(x)}=\pm {\beta_{n}}^{2},\\ \frac{\partial^{2}Y_{\theta \_m}(y)}{\partial y^{2}Y_{\theta \_m}(y)}=\pm {\mu_{m}}^{2},\\ \frac{\partial^{2}Z_{\theta \_n,m}(z)}{\partial x^{2}Z_{\theta \_n,m}(z)}=\pm {\gamma_{n,m}}^{2}, \end{array}\right.$$ (6)

where *β*_*n*_, *μ*_*m*_, and *γ*_*n*,*m*_ are eigenvalues. Taking the boundary conditions into consideration, the relation -*β*_*n*_^*2*^ *- μ*_*m*_^*2*^ +*γ*_*n*,*m*_^*2*^ = 0 appears to be more appropriate. The Fourier series form of the general solution can then be derived:

$$\left\{ \begin{array}{l} \theta =C_{1}z+C_{2}+\sum_{n=0}^{\infty }\sum_{m=0}^{\infty }C_{n,m}\cos(\beta_{n}x)\cos(\mu_{m}y)(sh(\gamma_{n,m}z)+C_{\gamma_{n,m}}ch(\gamma_{n,m}z)),\\ \beta_{n}=n\pi /L_{x},\;\mu_{m}=m\pi /L_{y},\;\gamma_{n,m}=\sqrt{{\beta_{n}}^{2}+{\mu_{m}}^{2}}, \end{array}\right.$$ (7)

where *C*_*1*_, *C*_*2*_, *C*_*n*,*m*_, and *C*_*γn*,*m*_ are coefficients; *n* and *m* are not simultaneously equal to zero in Fourier series. By substituting the general solution into the last thermal boundary condition of *θ*, *C*_*2*_ and *C*_*γn*,*m*_ can be derived [17].

Then, we can substitute the general solution into the boundary condition of *θ* at the top surface (*z = 0*). By further multiplying one set of $cos(\beta_{n}x)cos(\mu_{m}y)$ on both sides of the equation each time, and then calculating the integrals along the surface (*z* = 0), we can obtain *C*_*1*_ and *C*_*n*,*m*_ [17]. This is done based on the integration property of trigonometric functions.

Subsequently, the analytical solution of *θ* at any position within the prepreg layer can be expressed as an array product that is shown in (8), in which *C*_*1*_^*’*^ denotes the z-dependent coefficient that includes both *C*_*1*_ and *C*_*2*_; *C*_*qiu*,*i*_ denotes the (x, y)-dependent coefficient that includes *C*_*n*,*m*_; *C*^*’*^_*qiu*,*i*_ denotes the cell coefficient of *R*_*u*_*(x*,*y*,*z)*; *C*_*n*,*m*,*qiu*,*i*_ denotes the cell coefficient used to derive *C*^*’*^_*qiu*,*i*_; *q*_*Iu*_ denotes the array form of *q*_*iu*_*(x*,*y)*; and *N*_*e*_ denotes the number of *β*_*n*_ or *μ*_*m*_; and *N*_*1*_ denotes the number of discrete surface regions; *q*_*iu*,*i*_ denotes the approximate uniform heat flux in the *i*^*th*^ individual square region; *d*_*q*_ denotes the edge length of each region; and *δ*_*m*_ and *δ*_*n*_ denote constant coefficients. The analytical solution of *η* can be obtained in a similar manner.


{θ(x,y,z)=C1'(11⋯1)(qiu,1qiu,2⋮qiu,N1)+(Cqiu,1Cqiu,2⋯Cqiu,N1)(qiu,1qiu,2⋮qiu,N1)=(C'qiu,1C'qiu,2⋯C'qiu,N1)qIu=Rθ,u(x,y,z)qIuRθ,u(x,y,z)=(C'qiu,1C'qiu,2⋯C'qiu,N1)=(C1'cos(β1⋅x)cos(μ0⋅y)⋮cos(βNe⋅x)cos(μ0⋅y)cos(β0⋅x)cos(μ1⋅y)⋮cos(β0⋅x)cos(μNe⋅y)⋮cos(βNe⋅x)cos(μNe⋅y))T(11⋯1C1,0,qiu,1C1,0,qiu,2⋯C1,0,qiu,N1⋮⋮⋮CNe,0,qiu,1CNe,0,qiu,2⋯CNe,0,qiu,N1C0,1,qiu,1C0,1,qiu,2⋯C0,1,qiu,N1⋮⋮⋮C0,Ne,qiu,1C0,Ne,qiu,2⋯C0,Ne,qiu,N1⋮⋮⋮CNe,Ne,qiu,1CNe,Ne,qiu,2⋯CNe,Ne,qiu,N1)C1'=dq2*(−z+1Hd+Lin)kiLxLy[1+Hu(Lin+1Hd)]Cn,m,qiu,i=−(sh(γn,mz)+Cγn,mch(γn,mz))2(2−δn−δm)∬SQiu,icos(βn⋅x)cos(μm⋅y)dxdykiLxLy[γn,m+Huγn,mch(γn,mLin)+Hdsh(γn,mLin)γn,msh(γn,mLin)+Hdch(γn,mLin)] (8)

Further, the analytical solutions for both the upper and lower surfaces of each prepreg layer can be expressed as matrix equations:

$$\left\{ \begin{array}{l} T_{u}=\theta_{u}+\eta_{u}=R_{u,u}q_{Iu}+R_{u,d}q_{Id},\\ T_{d}=\theta_{d}+\eta_{d}=R_{d,u}q_{Iu}+R_{d,d}q_{Id}, \end{array}\right.$$ (9)

where *T*_*u*_ and *T*_*d*_ denote the temperature arrays of the upper and lower surfaces, respectively; *q*_*Id*_ denotes the array form of *q*_*id*_*(x*,*y)*; and *R*_*u*,*u*_, *R*_*u*,*d*_, *R*_*d*,*u*_, and *R*_*d*,*d*_ denote the coefficient matrices of thermal resistance related to thermal effects. Therefore, both surfaces of the prepreg layer are divided into discrete surface cells, and in each cell region the heat flux transferred is considered uniform. Particularly, the surface region attached to the metal layer has the same discretization layout as the metal layer to facilitate the consideration of the heat flux transferred from the metal layer.

Moreover, the assumed HTCs *h*_*u*_ and *h*_*d*_ shown in (2) and (3), may not accurately represent the actual conditions of some surface regions. For instance, direct thermal convection between covered regions of the PCB’s top surface and the environment is almost non-existent due to component presence. Moreover, if radiation heat transfer is taken into account, the equivalent HTCs of some surface regions may greatly deviate from the assumed average value. These unrealistic assumptions can be corrected by taking into account the respective compensated heat flux, which is related to the HTC difference and can be considered based on the superposition theorem of thermal effect.

When considering the compensated heat flux of each discrete region, the expression of the analytical solutions can be further expanded into the following form:

$$\left\{ \begin{array}{l} T_{u}=R_{u,u}q_{Iu}+R_{u,d}q_{Id}+R_{u,NMu}q_{ex,NMu}+R_{u,NMd}q_{ex,NMd},\\ T_{d}=R_{d,u}q_{Iu}+R_{d,d}q_{Id}+R_{d,NMu}q_{ex,NMu}+R_{d,NMd}q_{ex,NMd},\\ q_{ex,NMu}=M_{Hdif,NMu}T_{u},\\ q_{ex,NMd}=M_{Hdif,NMd}T_{d}, \end{array}\right.$$ (10)

where *M*_*Hdif*_ denotes the diagonal matrix representing the difference between *h*_*u*_ or *h*_*d*_ and the actual HTC of each discrete surface cell; *q*_*ex*_ denotes the array of compensated heat flux; the subscripts _*NMu*_ and _*NMd*_ refer to the upper and lower surface regions not attached to the metal layer, respectively; and the four matrices, *R*_*u*,*NMu*_, *R*_*u*,*NMd*_, *R*_*d*,*NMu*_, and *R*_*d*,*NMd*_, also denote the coefficient matrices related to thermal effect. For example, *R*_*u*,*NMu*_*q*_*ex*,*NMu*_ represents the thermal effect of *q*_*ex*,*NMu*_ on *T*_*u*_, and other coefficient matrices related to thermal effect function similarly. The compensated heat flux related to the region attached to the metal layer is going to be considered in the numerical approximation of heat diffusion in the metal layer.

When radiation heat transfer is considered, *M*_*Hdif*_ becomes dependent on the temperature distribution of the corresponding surface. In this case, iterations between the temperature distribution and the temperature-dependent HTC of each surface cell must be executed to search for the radiation-equivalent HTC of each cell and eventually identify the solutions.

To incorporate the numerical approximation of heat diffusion in the metal layer, the thermal effects of the metal-attached and non-metal-attached regions must be separately considered. Thus, the expressions of the analytical solution presented in (10) are transformed to the following form.

$$\left\{ \begin{array}{l} T_{u}=R_{u,Mu}q_{Mu}+R_{u,Md}q_{Md}+R_{u,NMu}(q_{NMu}+q_{ex,NMu})+R_{u,NMd}(q_{NMd}+q_{ex,NMd}),\\ T_{d}=R_{d,Mu}q_{Mu}+R_{d,Md}q_{Md}+R_{d,NMu}(q_{NMu}+q_{ex,NMu})+R_{d,NMd}(q_{NMd}+q_{ex,NMd}),\\ q_{ex,NMu}=M_{Hdif,NMu}T_{u},\\ q_{ex,NMd}=M_{Hdif,NMd}T_{d}, \end{array}\right.$$ (11)

where, *q*_*Mu*_ and *q*_*Md*_ denote the heat flux transferred to the upper and lower insulating surface regions attached to the metal layer, respectively; *q*_*NMu*_ and *q*_*NMd*_ denote the heat flux related to the regions not attached to the metal layer. Thus *q*_*Iu*_ is divided into *q*_*Mu*_ and *q*_*NMu*_, whereas *q*_*Id*_ is divided into *q*_*Md*_ and *q*_*NMd*_. Similar to the case of *R*_*u*,*NMu*_, the four matrices, *R*_*u*,*Mu*_, *R*_*u*,*Md*_, *R*_*d*,*Mu*_, and *R*_*d*,*Md*_, are also related to thermal effect.

### 2.2 Numerical approximation of heat transfer through the lateral sides of a PCB

**Fig 4:**
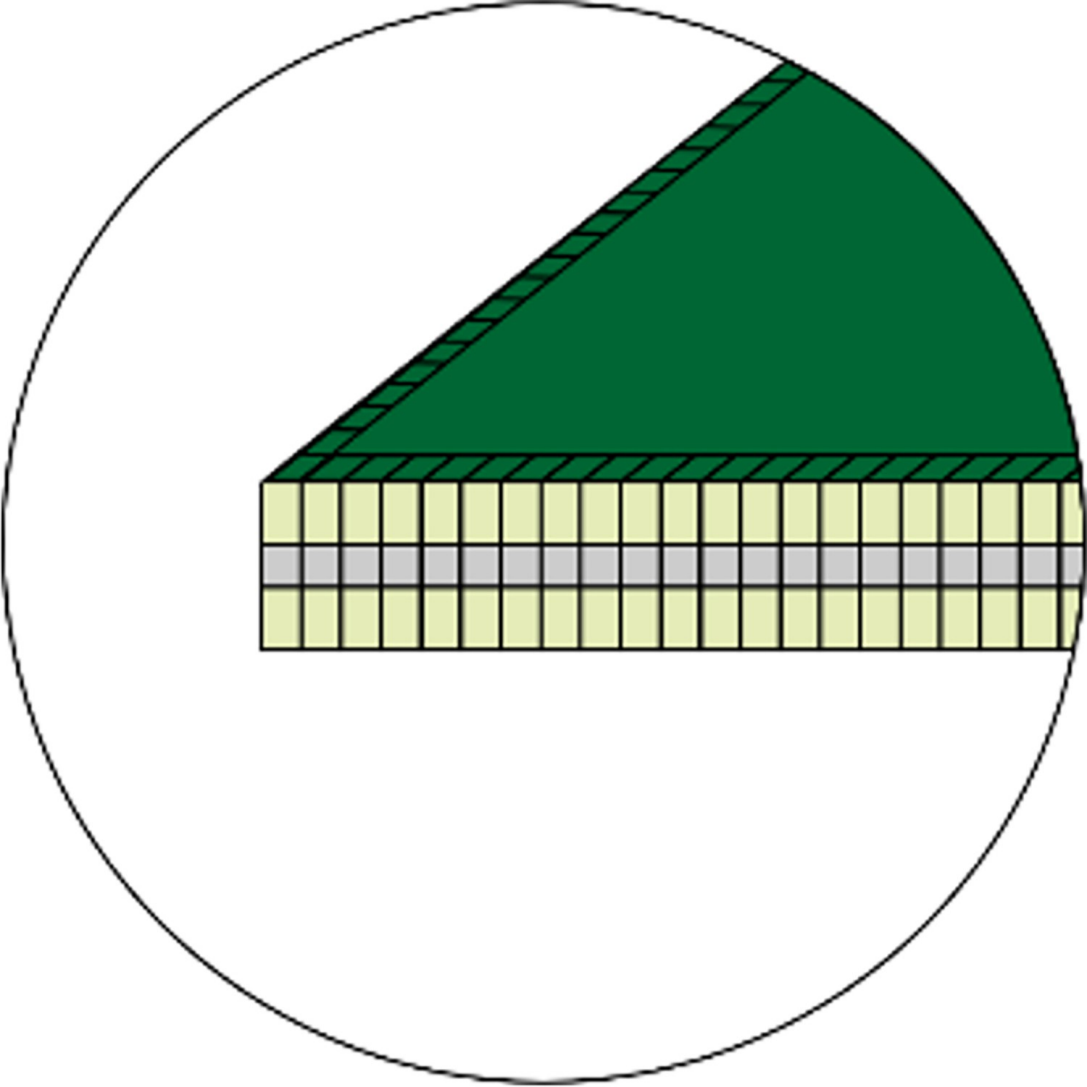
Diagram of the discrete cells along the lateral sides of the PCB.

Since the thickness of the prepreg layer or core layer is usually less than 2 mm and much smaller than the length and width of a PCB, the lateral sides of the insulating layer can only be discretized horizontally, as depicted in Fig 4. Similar to the boundary conditions presented in (2), heat transfer between the lateral sides of a PCB and the environment can also be approximately calculated by multiplying the HTC with the temperature of each individual lateral cell. For example, if a PCB is under the boundary condition of natural convection (still air) with an average HTC of *h*_*a*_, then the array of the heat flux on the lateral sides of the top prepreg layer, *q*_*le_tp*_, can be expressed as follows.


$$q_{le\_tp}=h_{a}T_{le\_tp},$$ (12)

where *T*_*le*_ denotes the array consisting of the temperatures of all individual cells on the lateral sides of the layer; the subscript _*tp*_ refers to the top prepreg layer.

To take *q*_*le*_ into consideration, its thermal effect should also be included in the analytical solutions shown in (11). But, depending on the boundary conditions given in (2), only the heat flux at the upper and lower surface of the prepreg layer can be taken into account in the analytical solutions. Thus, *q*_*le*_ or *T*_*le*_ must be related to the surface heat flux or temperature distributions of the corresponding insulating layer. One option is to average the temperatures of surface boundary cells to approximate those of the lateral cells:

$$T_{bc\_tp}=\frac{T_{bc,u\_tp}+T_{bc,d\_tp}}{2}=\frac{M_{bc,u\_tp}T_{u\_tp}+M_{bc,d\_tp}T_{d\_tp}}{2},$$ (13)

where *T*_*bc*,*u*_ and *T*_*bc*,*d*_ denote the temperature arrays of the boundary cells of the upper and lower surfaces of an insulating layer, respectively; *M*_*bc*,*u*_ and *M*_*bc*,*d*_ denote the coefficient matrices that facilitate the transformations between the temperature arrays; *T*_*bc*_ denotes the average of *T*_*bc*,*u*_ and *T*_*bc*,*d*_, and can further be used to derive *T*_*le*_:

$$\left\{ \begin{array}{l} T_{le\_tp}=M_{b\_tp}T_{bc\_tp}=\frac{M_{b\_tp}(M_{bc,u\_tp}T_{u\_tp}+M_{bc,d\_tp}T_{d\_tp})}{2}=M_{b,u\_tp}T_{u\_tp}+M_{b,d\_tp}T_{d\_tp},\\ M_{b,u\_tp}=\frac{M_{b\_tp}M_{bc,u\_tp}}{2},\\ M_{b,d\_tp}=\frac{M_{b\_tp}M_{bc,d\_tp}}{2}, \end{array}\right.$$ (14)

where *T*_*le*_ has four more elements compared to *T*_*bc*_, because of four more corner cells at the lateral sides compared to the surface boundary. *M*_*b*_ denotes the coefficient matrix that facilitates the transformation between *T*_*le*_ and *T*_*bc*_. *M*_*b*,*u*_ and *M*_*b*,*d*_ also denote the coefficient matrices that facilitate the transformation between these temperature arrays. Thus, (12) can be expressed as follows:

$$q_{le\_tp}=h_{a}T_{le\_tp}=h_{a}M_{b,u\_tp}T_{u\_tp}+h_{a}M_{b,d\_tp}T_{d\_tp}.$$ (15)

If there is no uniform HTC available for the lateral sides, the specified HTC for each lateral cell can also be included in a coefficient matrix like *M*_*Hdif*_ to derive *q*_*le*_:

$$q_{le\_tp}=M_{H,le\_tp}T_{le\_tp}=M_{H,le\_tp}M_{b,u\_tp}T_{u\_tp}+M_{H,le\_tp}M_{b,d\_tp}T_{d\_tp}.$$ (16)

Similarly, heat transfer through the lateral sides of the core layer and the bottom prepreg layer can also be approximately described as follows:

$$q_{le\_core}=M_{H,le\_core}T_{le\_core}=M_{H,le\_core}M_{b,d\_tp}T_{d\_tp}+M_{H,le\_core}M_{b,u\_bp}T_{u\_bp},$$ (17)

$$q_{le\_bp}=M_{H,le\_bp}T_{le\_bp}=M_{H,le\_bp}M_{b,u\_bp}T_{u\_bp}+M_{H,le\_bp}M_{b,d\_bp}T_{d\_bp},$$ (18)

where the subscripts _*bp*_ and _*core*_ refer to the bottom prepreg layer and middle core, respectively. Further, each product term in the expression of *q*_*le*_ can be viewed as part of the compensated heat flux and included in *q*_*ex*_ of the corresponding layer-surface. For example, those two terms related to *T*_*d_tp*_ can be included in *q*_*ex*,*NMd_tp*_:

qex,NMd_tp=MHdif,NMd_tpTd_tp=MHdif,NMd_tp'Td_tp−(MH,le_tpMb,d_tpTd_tp+MH,le_coreMb,d_tpTd_tp), (19)

where *q*_*ex*,*NMd*_ and *M*_*Hdif*,*NMd*_ maintain their respective meanings, as defined previously for (11); *M*_*Hdif*,*NMd_tp*_*’T*_*d_tp*_ refers to other compensated heat flux related to *T*_*d_tp*_ except the heat flux through the lateral sides. The two terms inside parentheses are subtracted, because *q*_*le*_ is considered positive when heat is transferred from the lateral sides of the insulating layer to the environment. Therefore, *q*_*le*_ can be approximated as an element of *q*_*ex*_, and its thermal effect can thus be taken into account.

### 2.3 Numerical approximation of heat diffusion in metal-foil layers

In (11), all the heat flux passing through each prepreg-layer surface is depending on both the heat flux transferred to the metal layer and the following process of heat diffusion within the metal layer. A metal layer may comprise multiple metal traces of various patterns. Thus, the aforementioned Fourier-series based analytical solution is unsuitable for modeling the metal layer. Instead, Finite volume method (FVM) [26] can be used to discretize the metal layers and approximate internal heat diffusion. FVM is typically based on simplifying the integration of the differential equation that corresponds to each discrete element [26]. The iterative electro-thermal modeling method for analyzing IR-drop and Joule heating in metal layers was also partly based on the FVM [16].

**Fig 5:**
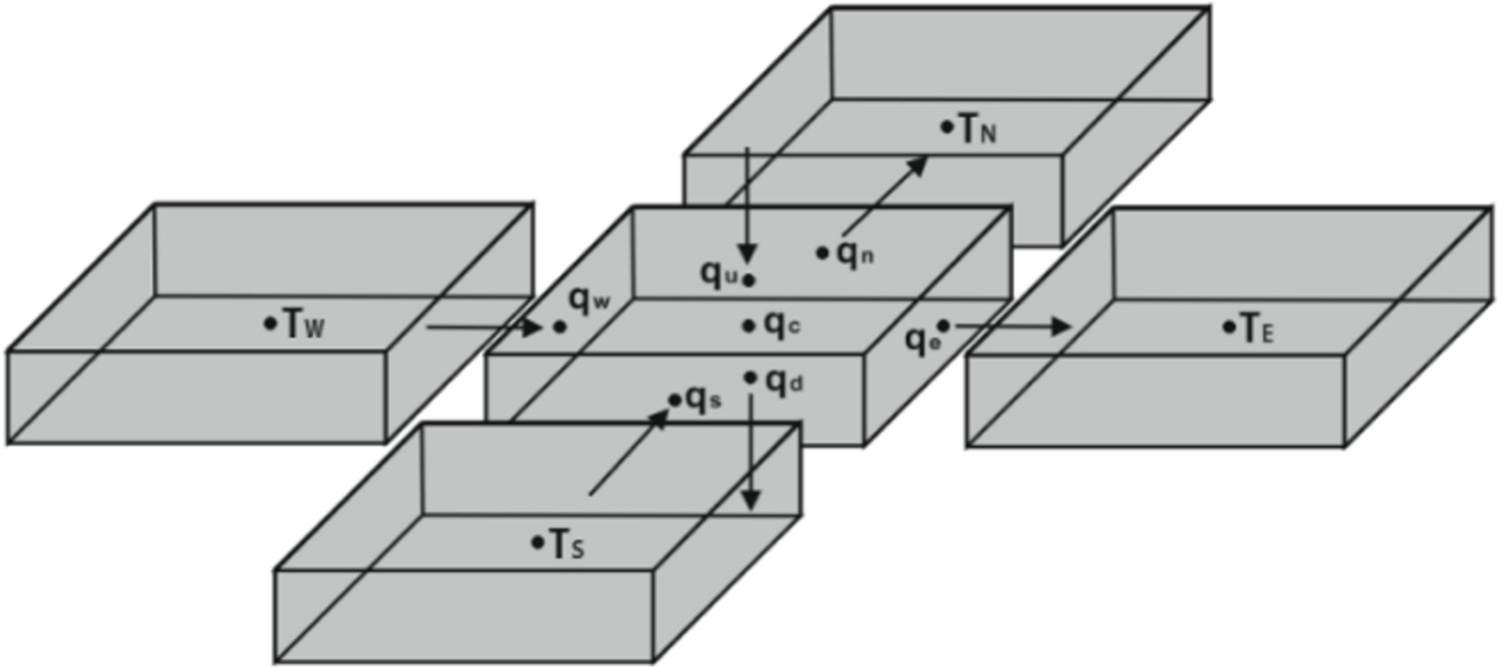
Schematic diagram of heat conduction through discrete cells in the metal layer.

As the thickness of the metal layer usually falls in the tens of microns, the vertical temperature variation through the layer can be ignored. Thus, the metal layer can be discretized only in the lateral direction. Consequently, the temperature distribution of the metal layer also mirrors that of the attached surface region of the prepreg layer. Square discrete cells, as shown in Fig 5, can be easily obtained by analyzing the pixel information of the layout map, and are also convenient to generate the multigrid.

The governing equation for steady-state heat diffusion of a discrete metal cell has the form of Poisson’s equation:

$$\frac{\partial^{2}T}{\partial x^{2}}+\frac{\partial^{2}T}{\partial y^{2}}+\frac{\partial^{2}T}{\partial z^{2}}+\frac{q_{J}}{k_{m}}=0,$$ (20)

where *q*_*J*_ denotes the Joule heating rate, and *k*_*m*_ denotes the thermal conductivity of the metal layer.

Using Gauss’s divergence theorem, the volume integration of (20) can be converted into closed-surface integration. For example, when considering the central discrete cell depicted in Fig 5, the integration over its six surfaces can be expressed as follows:

$$\int \left(q_{e}dA-q_{w}dA+q_{n}dA-q_{s}dA+q_{u}dA-q_{d}dA\right)=-q_{c}{d_{c}}^{2}d_{m},$$ (21)

where *d*_*c*_ denotes the cell length; *A* denotes the area variable; *d*_*m*_ denotes the thickness; *q*_*c*_ denotes the Joule heating rate; and *q*_*e*_, *q*_*w*_, *q*_*n*_, and *q*_*s*_ denote the heat flux normal to the four lateral surfaces; *q*_*u*_ and *q*_*d*_ denote the heat flux transferred into the upper side and out from the lower side of the metal cell, respectively. Thus, corresponding to the first metal layer at the top side of the PCB, *q*_*u*_ can represent the compensated heat flux due to the possible non-realistic assumption of *h*_*u*_ or the heat flux transferred from the component.

By employing Taylor series truncation technique with 2^nd^ order precision [26], (21) can be simplified further to (22). The temperatures of the four neighboring cells depicted in Fig 5 are denoted by *T*_*E*_, *T*_*W*_, *T*_*N*_, and *T*_*S*_, respectively. *T*_*c*_ denotes the temperature of the central cell. *ψ*_*Nv*,*z*_ denotes the discrete vertical thermal resistance of the via in the insulating layer, and *T*_*c*,*dv*_ denotes the temperature of the connected via cell in the other metal layer that is attached to the same insulating layer.


$$\begin{array}{l} k_{m}d_{c}d_{m}(\frac{T_{c}-T_{E}}{d_{c}}-\frac{T_{W}-T_{c}}{d_{c}}+\frac{T_{c}-T_{N}}{d_{c}}-\frac{T_{S}-T_{c}}{d_{c}})+\frac{T_{c}-T_{c,dv}}{\psi_{Nv,z}}=q_{c}{d_{c}}^{2}d_{m}+q_{u}{d_{c}}^{2}-q_{d}{d_{c}}^{2}\\ \Rightarrow (\frac{4k_{m}d_{m}}{{d_{c}}^{2}}+\frac{1}{\psi_{Nv,z}})T_{c}-\frac{k_{m}d_{m}}{{d_{c}}^{2}}(T_{E}+T_{W}+T_{N}+T_{S})-\frac{T_{c,dv}}{\psi_{Nv,z}{d_{c}}^{2}}=q_{c}d_{m}+q_{u}-q_{d}. \end{array}$$ (22)

The upper approximate discrete equation of heat diffusion for each group of adjacent metal cells can be gathered in a matrix equation. For example, corresponding to each pair of metal layers attached to a prepreg layer, the approximate matrix equations of heat diffusion can be derived as follows:

$$\left\{ \begin{array}{l} M_{Mu}T_{Mu}-M_{Vd}T_{Md}=q_{MJ,Mu}-q_{Mu},\\ M_{Md}T_{Md}-M_{Vu}T_{Mu}=q_{MJ,Md}-q_{Md},\\ q_{MJ,Mu}=q_{c,Mu}+q_{u,Mu}+M_{Hdif,Mu}T_{u},\\ q_{MJ,Md}=q_{c,Md}+q_{d,Md}+M_{Hdif,Md}T_{d}, \end{array}\right.$$ (23)

where the subscripts, _*Mu*_ and _*Md*_, refer to the upper and lower metal layers, respectively; the matrix, *M*_*Mu*_ or *M*_*Md*_, is composed of the coefficients of *T*_*E*_, *T*_*W*_, *T*_*N*_, *T*_*S*_, and *T*_*c*_ in each discrete equation of the corresponding metal layer; and the matrix, *M*_*Vu*_ or *M*_*Vd*_, is composed of the corresponding coefficient of *T*_*C*,*dv*_, in each discrete equation of the upper or lower metal layer; the terms, *M*_*Hdif*,*Mu*_*T*_*u*_ and *M*_*Hdif*,*Md*_*T*_*d*_, depend on the compensated heat flux of the metal-layer regions; *q*_*c*_,_*Mu*_ and *q*_*u*_,_*Mu*_ include *q*_*c*_*d*_*m*_ *and q*_*u*_ of each metal cell in the upper metal layer, respectively, whereas *q*_*c*_,_*Md*_ and *q*_*d*_,_*Md*_ are defined for the lower layer; and *q*_*Mu*_ or *q*_*Md*_ includes *q*_*d*_ or *q*_*u*_ of the corresponding metal layer, thereby representing the heat flux flowing to the attached prepreg layer and determining the analytical solutions as shown previously in (11).

To reduce the number of variables, *T*_*Mu*_ and *T*_*Md*_ are expressed using *T*_*u*_ and *T*_*d*_, respectively:

$$\left\{ \begin{array}{l} T_{Mu}=C_{M,u}T_{u},\\ T_{Md}=C_{M,d}T_{d}. \end{array}\right.$$ (24)

where *C*_*M*,*u*_ and *C*_*M*,*d*_ denote the coefficient matrices related to the conversion.

Eventually, the matrix equations that describe heat diffusion in two metal layers can be linked to the analytical solution of the attached prepreg layer as follows:

$$\left\{ \begin{array}{l} T_{u}=R_{u,Mu}q_{Mu}+R_{u,Md}q_{Md}+R_{u,NMu}(q_{NMu}+M_{Hdif,NMu}T_{u})+R_{u,NMd}(q_{NMd}+M_{Hdif,NMd}T_{d}),\\ T_{d}=R_{d,Mu}q_{Mu}+R_{d,Md}q_{Md}+R_{d,NMu}(q_{NMu}+M_{Hdif,NMu}T_{u})+R_{d,NMd}(q_{NMd}+M_{Hdif,NMd}T_{d}),\\ M_{MCu}T_{u}-M_{VCd}T_{d}=q_{c,Mu}+q_{u,Mu}+M_{Hdif,Mu}T_{u}-q_{Mu},\\ M_{MCd}T_{d}-M_{VCu}T_{u}=q_{c,Md}+q_{d,Md}+M_{Hdif,Md}T_{d}-q_{Md}, \end{array}\right.$$ (25)

$$\left\{ \begin{array}{l} M_{MCu}=M_{Mu}C_{M,u},\\ M_{MCd}=M_{Md}C_{M,d},\\ M_{VCu}=M_{Vu}C_{M,u},\\ M_{VCd}=M_{Vd}C_{M,d}. \end{array}\right.$$ (26)

Therefore, heat diffusion in metal layers can also be considered as an additional thermal boundary condition of the attached prepreg layer.

### 2.4 “Thermally-thick” approximation of the core layer and the test solver

For easily coupling the two “double-sided PCB” units in a four-layer PCB, the middle core layer is approximated as a “thermally thick” layer. Because there is a significant difference in thermal conductivity between the epoxy-based core layer and the attached copper layers. In a typical FR-4 PCB, the electrodeposited (ED) copper-foil layers of 20~50μm thick have a thermal conductivity of around 316 W/mK [27], much higher than that of the epoxy-based core layer, 0.3 W/mK (according to the material library of COMSOL). Hence, the heat flow through the core can be derived using the surface temperature distributions and discrete thermal resistances of the core.

In this way, the double-sided heat-transfer matrix equations shown in [25] for each group of two metal layers and one prepreg layer can be interconnected, forming the group of equations as depicted in [27] for the four-layer PCB structure. The first four equations correspond to the double-sided structure of the top prepreg layer that is denoted by the subscript _*tp*_, and the final four equations with the subscript _*bp*_ correspond to that of the bottom prepreg layer. The heat flux transferred between two double-sided structures denoted by *q*_*u*,*Md_tp*_, *q*_*NMd_tp*_, *q*_*u*,*Mu_bp*_, and *q*_*NMu_bp*_, are approximately related to *T*_*u_bp*_ and *T*_*d_tp*_, which also represent the temperature distributions of the corresponding core surfaces, respectively. Further, *T*_*u_bp*_ and *T*_*d_tp*_ are also used to approximately calculate the temperature distributions of the lateral sides of the core layer. Thus, heat transfer through the lateral sides of the core layer can also be taken into account using the approach of compensated heat flux. *C*_*Md_tp*_, *C*_*NMd_tp*_, *C*_*Mu_bp*_, and *C*_*NMu_bp*_ denote the coefficient matrices corresponding to the discrete vertical thermal resistances of the core layer. The remaining arrays, coefficient matrices, and subscripts have been defined previously.


$$\left\{ \begin{array}{l} T_{u\_tp}=R_{u\_tp,Mu\_tp}q_{Mu\_tp}+R_{u\_tp,Md\_tp}q_{Md\_tp}+R_{u\_tp,NMu\_tp}(q_{NMu\_tp}+M_{Hdif,NMu\_tp}T_{u\_tp})+R_{u\_tp,NMd\_tp}(q_{NMd\_tp}+M_{Hdif,NMd\_tp}T_{d\_tp}),\\ T_{d\_tp}=R_{d\_tp,Mu\_tp}q_{Mu\_tp}+R_{d\_tp,Md\_tp}q_{Md\_tp}+R_{d\_tp,NMu\_tp}(q_{NMu\_tp}+M_{Hdif,NMu\_tp}T_{u\_tp})+R_{d\_tp,NMd\_tp}(q_{NMd\_tp}+M_{Hdif,NMd\_tp}T_{d\_tp}),\\ M_{MCu\_tp}T_{u\_tp}-M_{Vcd\_tp}T_{d\_tp}=q_{c,Mu\_tp}+q_{u,Mu\_tp}+M_{Hdif,Mu\_tp}T_{u\_tp}-q_{Mu\_tp},\\ M_{MCd\_tp}T_{d\_tp}-M_{Vcu\_tp}T_{u\_tp}=q_{c,Md\_tp}+q_{d,Md\_tp}+M_{Hdif,Md\_tp}T_{d\_tp}-q_{Md\_tp},\\ q_{d,Md\_tp}=C_{Md\_tp}(T_{u\_bp}-T_{d\_tp}),\\ q_{NMd\_tp}=C_{NMd\_tp}(T_{u\_bp}-T_{d\_tp}),\\ q_{u,Mu\_tp}=C_{Mu\_bp}(T_{d\_tp}-T_{u\_bp}),\\ q_{NMu\_bp}=C_{NMu\_bp}(T_{d\_tp}-T_{u\_bp}),\\ T_{u\_bp}=R_{u\_bp,Mu\_bp}q_{Mu\_bp}+R_{u\_bp,Md\_bp}q_{Md\_bp}+R_{u\_bp,NMu\_bp}(q_{NMu\_bp}+M_{Hdif,NMu\_bp}T_{u\_bp})+R_{u\_bp,NMd\_bp}(q_{NMd\_bp}+M_{Hdif,NMd\_bp}T_{d\_bp}),\\ T_{d\_bp}=R_{d\_tp,Mu\_bp}q_{Mu\_bp}+R_{d\_bp,Md\_bp}q_{Md\_bp}+R_{d\_bp,NMu\_bp}(q_{NMu\_bp}+M_{Hdif,NMu\_bp}T_{u\_bp})+R_{d\_bp,NMd\_bp}(q_{NMd\_bp}+M_{Hdif,NMd\_bp}T_{d\_bp}),\\ M_{MCu\_bp}T_{u\_bp}-M_{Vcd\_bp}T_{d\_bp}=q_{c,Mu\_bp}+q_{u,Mu\_bp}+M_{Hdif,Mu\_bp}T_{u\_bp}-q_{Mu\_bp},\\ M_{MCd\_bp}T_{d\_bp}-M_{Vcu\_bp}T_{u\_bp}=q_{c,Md\_bp}+q_{d,Md\_bp}+M_{Hdif,Mu\_bp}T_{d\_bp}-q_{Md\_bp}. \end{array}\right.$$ (27)

**Fig 6:**
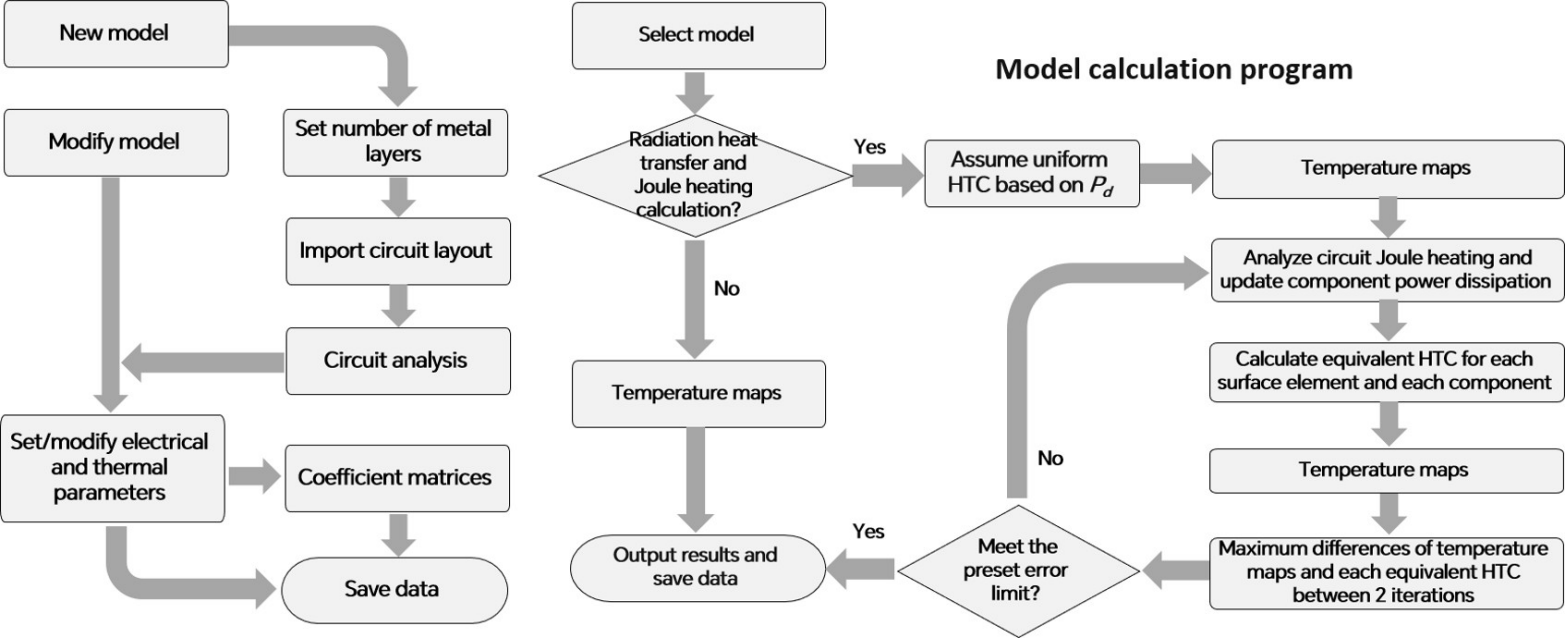
Program flow chart of the test solver. (a) Model construction program. (b) Model calculation program.

Based on the modeling method, a test solver was developed in MATLAB. The test solver consists of two main parts, as shown in Fig 6. In the “Model construction program”, all the coefficient matrices presented in (27) are generated. When modifying a model, the coefficient matrices should only be recalculated if there are changes in the structural parameters, such as the dimensions, or thermal parameters, like the assumed HTCs associated with the analytical solutions.

In the “Model calculation program”, the upper equations are solved using a single left-division operation. If there are boundary conditions of temperature-dependent heat flux defined in the model, such as radiation heat transfer, a new group of heat-flux equivalent HTCs can be derived and used for a new round of calculations. Such iterations continue until the preset acceptable errors in both the temperature maps and temperature-dependent HTCs are reached. The total Joule heating in traces and vias can also be iteratively calculated based on the temperature-dependent distribution of electric potential, and is then used to update the power dissipation of components based on the total power dissipation of the circuit (*P*_*d*_).

## 3. The model of one four-layer stack

### 3.1 Modeling parameters

**Fig 7:**
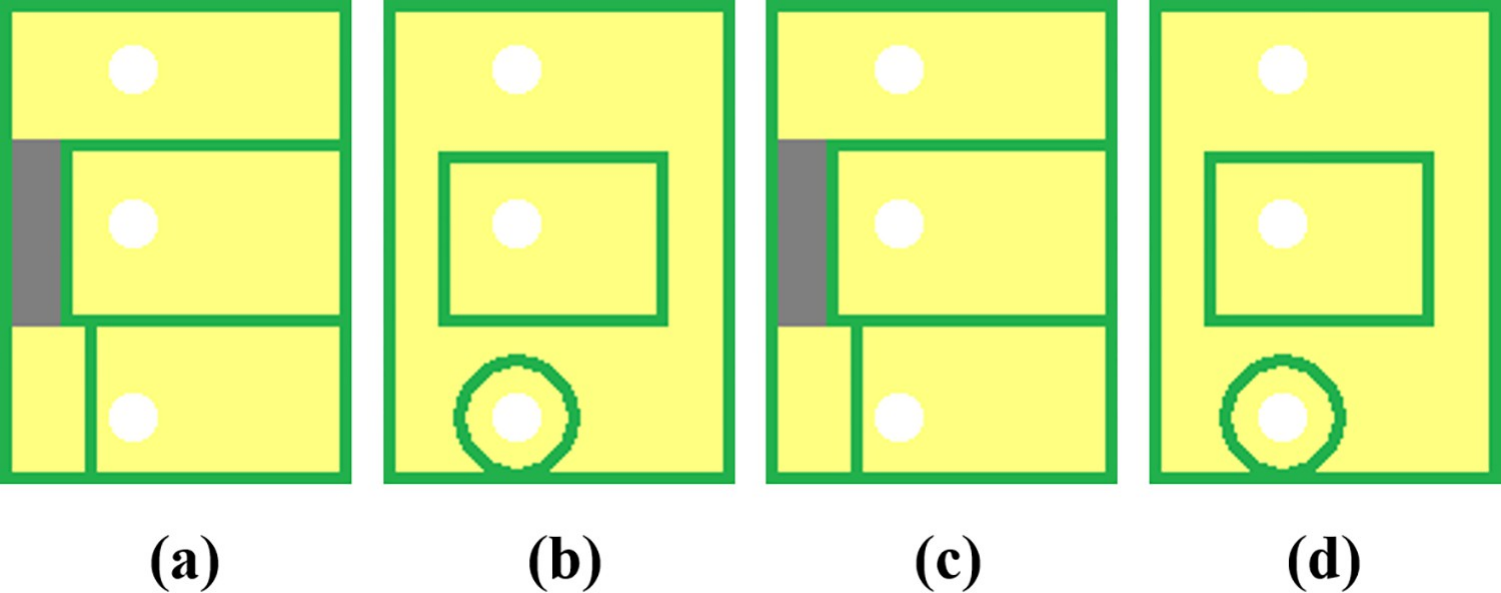
Metal layer layouts of Structure A. (a) first layer. (b) second layer. (c) third layer. (d) fourth layer.

A four-layer stack—Structure A—as illustrated in Fig 7 was modeled using both the test solver and COMSOL Multiphysics. The stack has dimensions of 29 mm × 40 mm. Each prepreg layer was set to be 0.639 mm thick. The copper thickness of the top and bottom layers was set to 35μm, while that of two middle layers was set to 30μm. This setting was based on the structure parameter of a PCB for generating DC 3.3V, which was also thermally modeled and will be introduced in the next section. The thickness of the core layer, *Th*_*core*_, was set to either 0.15 mm or 0.55 mm during modeling to study its impact on accuracy.

The thermal conductivity of the prepreg material was assumed to be equal to that of the epoxy-based core. This is because “prepreg” only indicates that the epoxy resin has dried but not yet hardened. The stack was assumed to be a common FR-4 type. Thus, as mentioned in the previous section, the thermal conductivity of the insulating layers was taken to be 0.3 W/mK, and that of the copper foil was taken to be 316 W/mK [27].

There are three copper vias in the structure. The via in the middle only connects the first and second metal-foil layers, whereas the other two vias run through all the layers. The thickness of each via wall was taken to be 18 μm. The heat was assumed to be uniformly transferred into the grey region in the first layer, and *P*_*d*_ was set to from 0.2W to 1 W to investigate the stability of the modeling accuracy. The thermal boundary conditions were assumed to be both natural convection (still air) with an equivalent HTC of 10 W/m^2^K [1] and radiation heat transfer. The radiation emissivity of all the surfaces was set to 0.9, an approximate value corresponding to the surfaces of the PCB and ICs [2,30,31]. The environment was assumed to be a blackbody at 20°C. Two modeling cases as shown in Table 1 were compared to investigate both the influence on the accuracy when heat transfer through the lateral sides of the PCB was not included, and that of the numerical approximation of the lateral sides.

**Table 1:** Modeling condition definition related to the model of Structure A.

Modeling case in the Test Solver	Case-1	Case-2
Heat transfer through the lateral sides of the PCB	Not included	included

**Fig 8:**
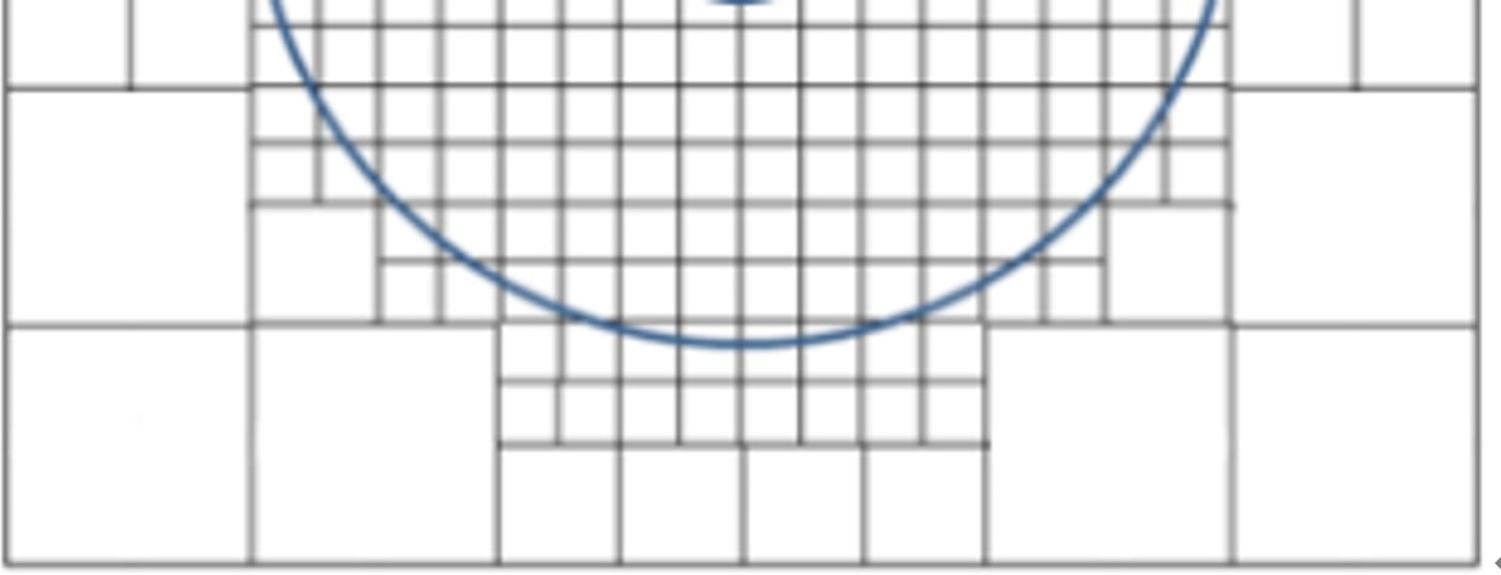
Schematic diagram of discrete cells under a three-level multigrid.

Using the test solver, 2D layout maps shown in Fig 7 can be analyzed by first considering each pixel as a discrete cell. The pixel distribution with a resolution of 116 × 160 can be considered as a one-level grid with the unit cell length of 0.25 mm. In order to model the stack more efficiently, a three-level multigrid was used to discretize metal layers and surfaces. Fig 8 shows a schematic diagram of the discrete cells generated under the three-level multigrid. The generation of the multi-level discrete cells can be realized simply by unifying the neighboring cells together [17]. To guarantee the accuracy of the hot-spot region, the coarser grids were not used for the heating region. Finally, the number of discrete cells of each layer surface was reduced to less than 5000, thus decreasing the total operation burden significantly:

The number of the eigenvalue, *β*_*n*_, is equal to that of μm and is denoted by Ne. Ne was finally set to 300, resulting in about 9 x 10^4^ Fourier series in each analytical solution. Further increasing Ne did not have an appreciable impact on the modeling accuracy. During the iterations for calculating radiation heat transfer, the accepted errors of the equivalent HTC and temperature of each discrete cell were set to 0.001W/m^2^K and 0.001°C, respectively.

**Fig 9:**
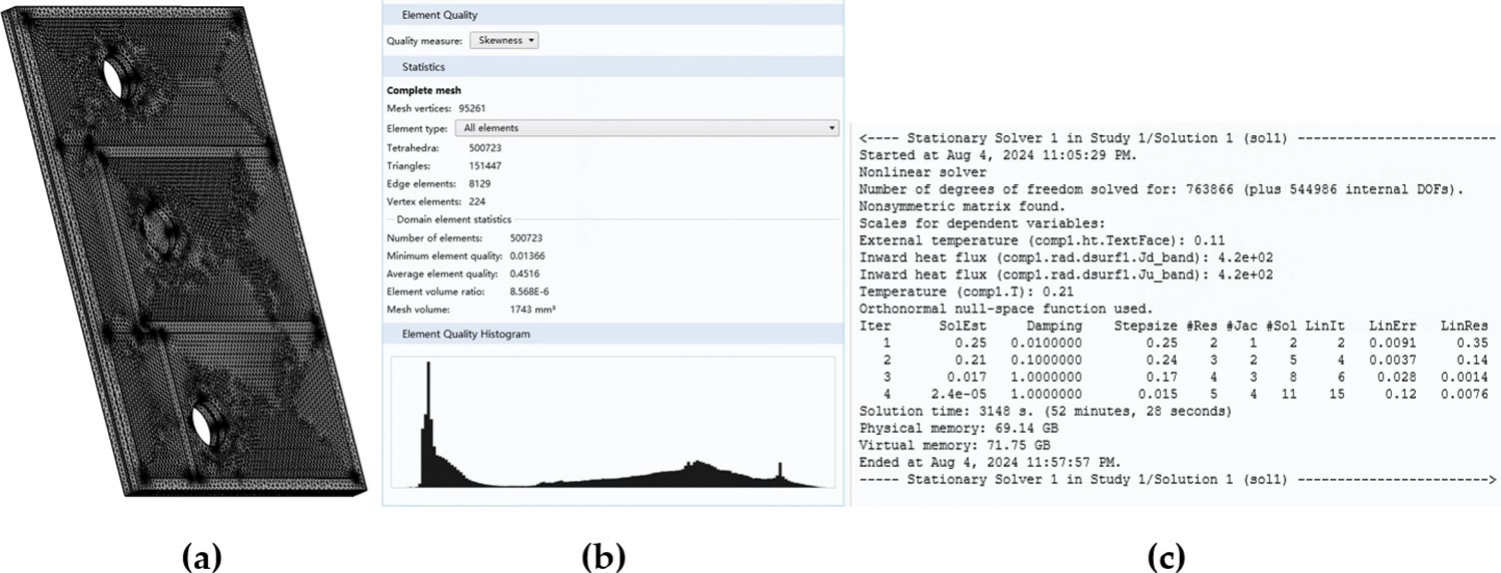
Mesh and simulation parameters of the COMSOL model of Structure A with Th_core_ = 0.15 mm under Case-2. (a) Mesh view. (b) Mesh quality under “Fine” level. (c) Simulation parameters.

In order to verify the modeling results, two 3D models were constructed using COMSOL 5.5, corresponding to the two different *Th*_*core*_ values. The mesh view and parameters of the COMSOL model with *Th*_*core*_ = 0.15 mm are shown in Fig 9. The "Heat Transfer in Solids" module and the "Surface-to-Surface Radiation" module were selected to identify the solution.

### 3.2 Modeling results

**Fig 10:**
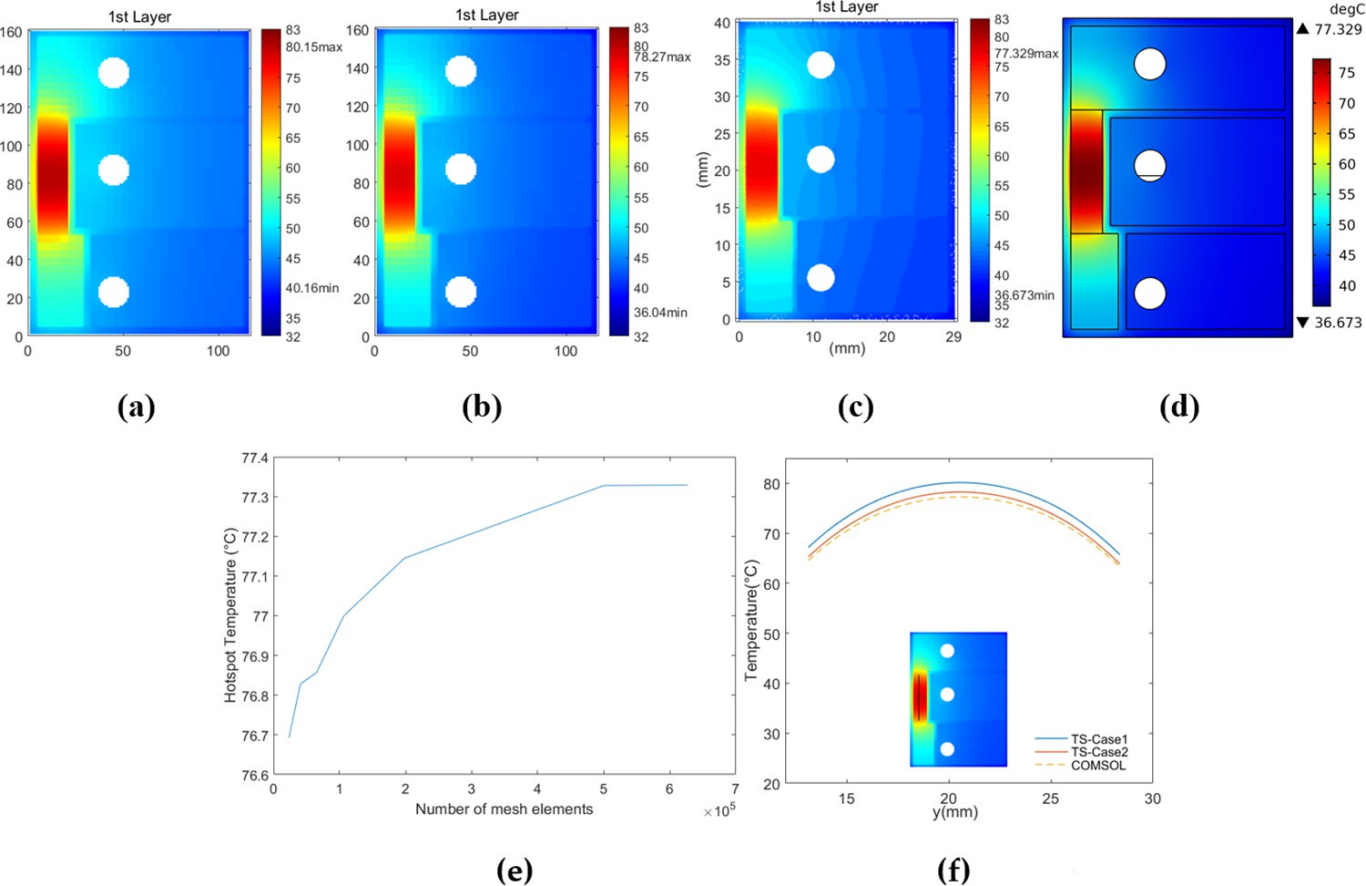
Temperature maps of the first-layer surface of Structure A with Th_core_ = 0.15 mm. (a) Case-1. (b) Case-2. (c) Figure with COMSOL data. (d) COMSOL output. (e) COMSOL convergence curve of the hotspot temperature under Case-2. (f) Temperature profiles along the marking line.

**Fig 11:**
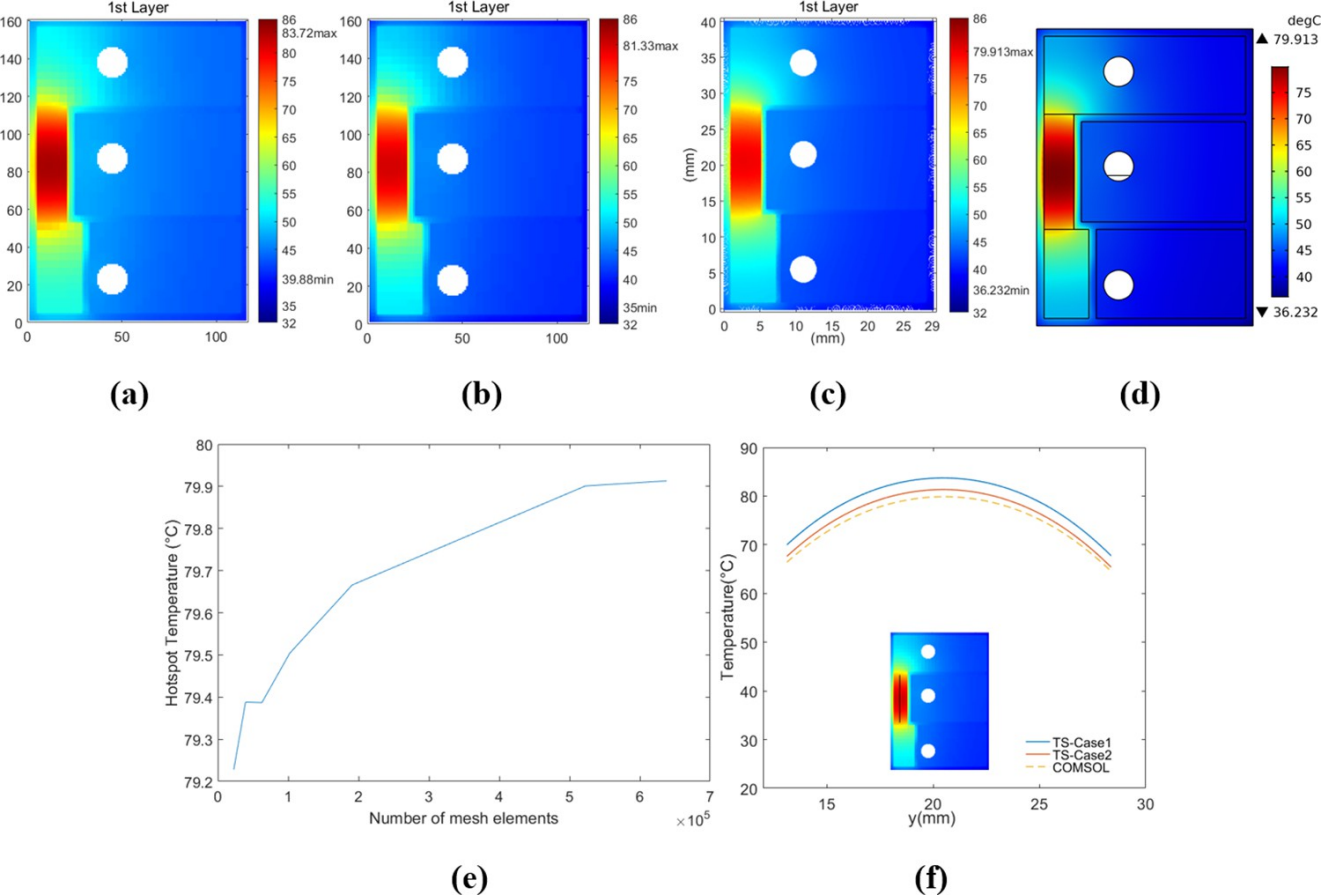
Temperature maps of the first-layer surface of Structure A with Th_core_ = 0.55 mm. (a) Case-1. (b) Case-2. (c) Figure with COMSOL data. (d) COMSOL output. (e) COMSOL convergence curve of the hotspot temperature under Case-2. (f) Temperature profiles along the marking line.

In Fig 10, temperature maps of the first layer surface from both the Test Solver (TS) and COMSOL models with *Th*_*core*_ = 0.15 mm and *P*_*d*_ = 1W are displayed, along with a comparison of temperature profiles along the marking line (x = 2.625mm, y = 13.125mm~28.375mm) in the hotspot region. The term “first layer surface” refers to the prepreg-layer surface that is attached to the first metal layer. Similar comparisons for models with *Th*_*core*_ = 0.55 mm and *P*_*d*_ = 1W are depicted in Fig 11. Obviously, under the assumed thermal boundary conditions, ignoring heat transfer of the lateral sides in Case-1 produces significant errors. This is because the amount of heat transfer from the lateral sides of the PCB reached about 7.1% and 8.9% of the total heating power for the two structures, respectively.

**Fig 12:**
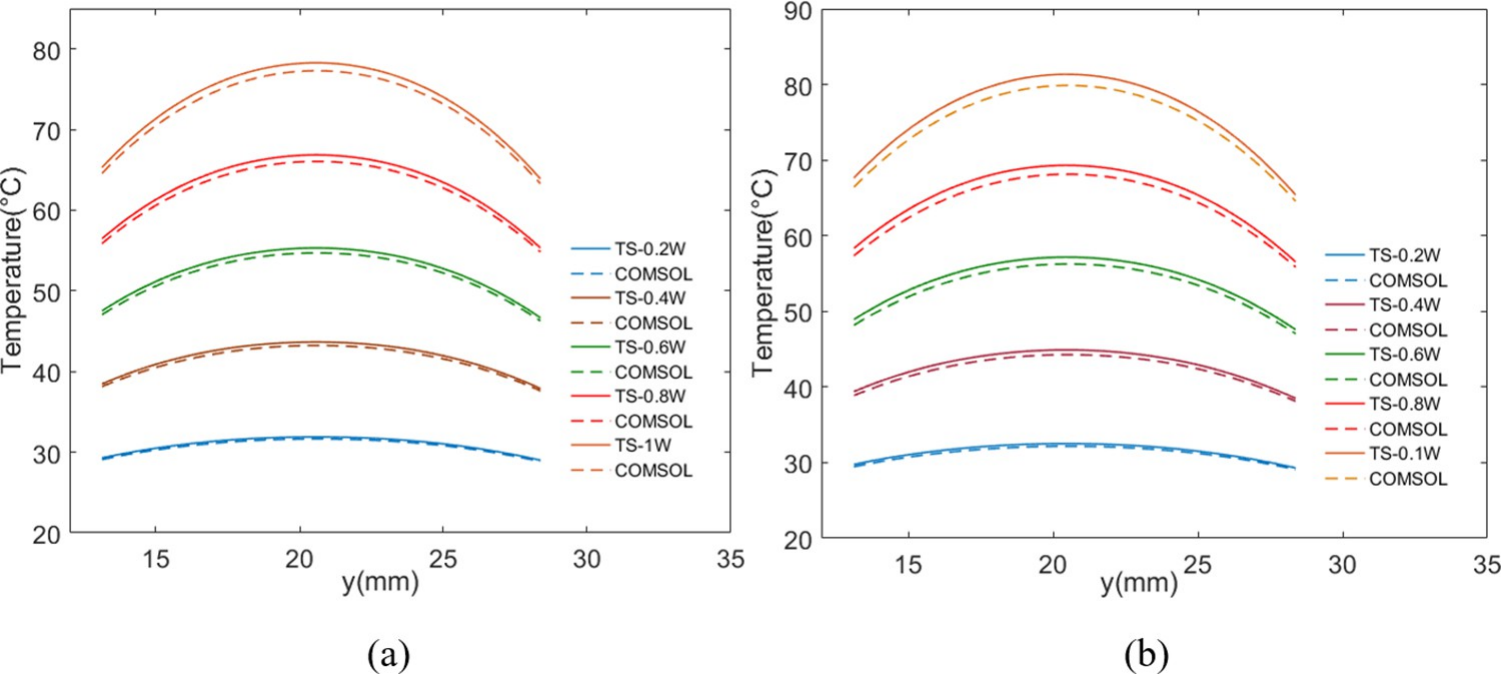
Comparison of temperature curves of the marking hot region between Case-2 and COMSOL models with different P_d_. (a) Th_core_ = 0.15 mm. (b) Th_core_ = 0.55 mm.

Comparisons under Case-2 with other heating power were also conducted, and the corresponding temperature curves of the marking hot region are depicted in Fig 12. For models with *Th*_*core*_ = 0.15 mm, the average modeling error rates of the marking hot region were around 1.8%, whereas those for *Th*_*core*_ = 0.55 mm were around 2.5%. Hence, the “thermally thick” approximation of the core layer didn’t exert a significant impact on the accuracy, which can be attributed to the much more effective lateral-heat-diffusion within the copper layers attached to core.

**Fig 13:**
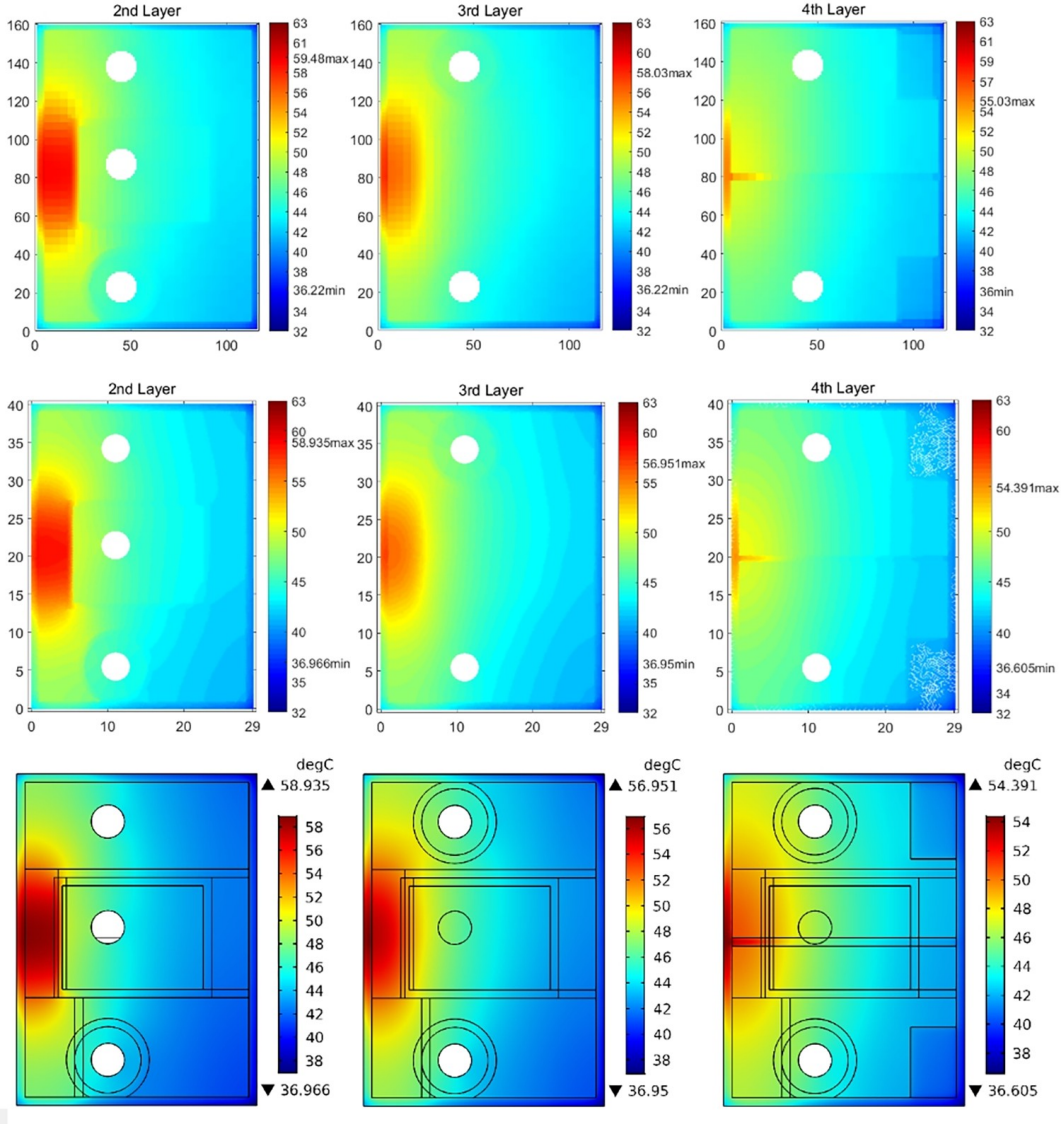
Comparison of temperature maps of other three layer surfaces between Case-2 (top figures) and COMSOL (middle: figures with COMSOL data, and bottom: COMSOL output) models with *Th*_*core*_ = 0.15 mm and *P*_*d*_ = 1W.

In Fig 13, the comparison of temperature maps of other three layer surfaces are illustrated for the Case-2 model with *Th*_*core*_ = 0.15 mm and *P*_*d*_ = 1W. Therefore, the modeling results of the test solver were testified to be relatively consistent with those obtained using COMSOL.

The models were run on a computer composed of an Intel 6138 CPU with 40 cores, 256GB memory, and 1TB solid-state drive. Both the preset accepted errors under Case-1 and Case-2 were reached around 10 iterations. The operation time of each TS model was less than 9 minutes, whereas the COMSOL model in each case required around 50 minutes to achieve an approximately converged hotspot temperature with at least 7.6 x 10^5^ degrees of freedom. This was based on the results from varying different numbers of mesh elements. The much longer running time of the COMSOL models may be attributed to the combined computation and potential iterations between the "Heat Transfer in Solids" module and the "Surface-to-Surface Radiation" module. Secondly, in the COMSOL models with approximately converged hotspot temperatures, the total number of mesh elements was higher than 5 x 10^5^, whereas that of metal layers was only less than 2 x 10^5^. Hence, probably most of the computing resources were used to determine heat transfer and temperature distribution related to the insulating layers, rather than the metal layers which served as the primary heat diffusion path.

## 4. A PCB model

### 4.1 PCB parameters

**Fig 14:**
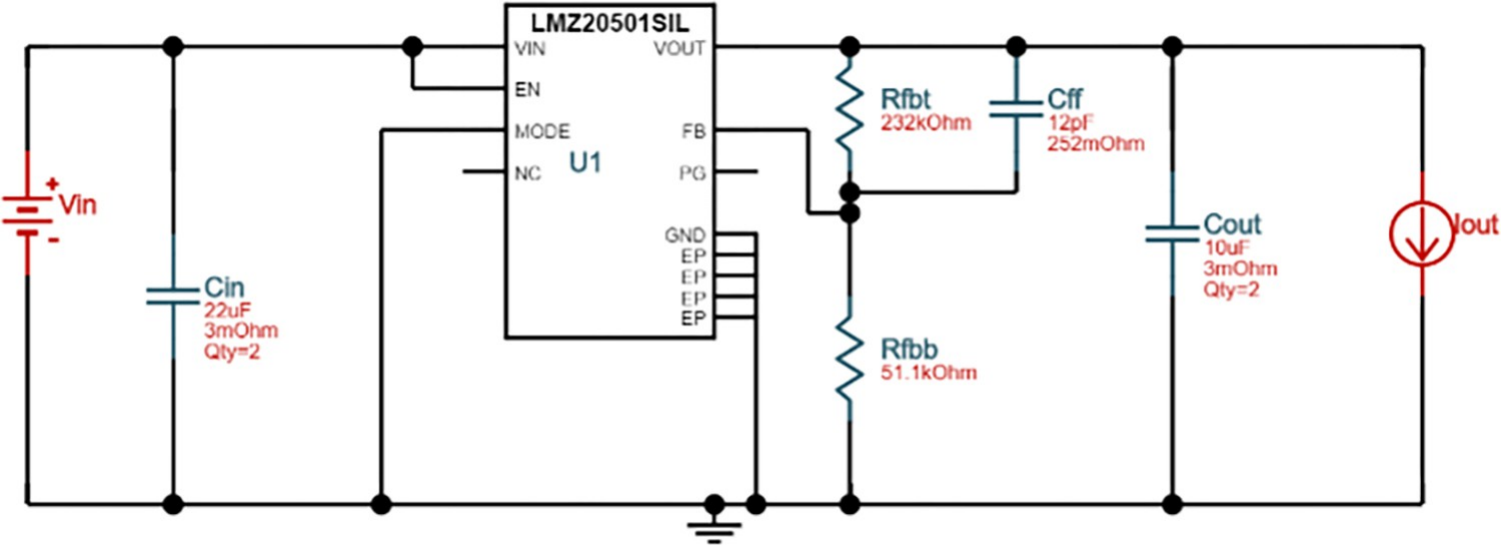
Circuit schematic of the PCB.

**Fig 15:**
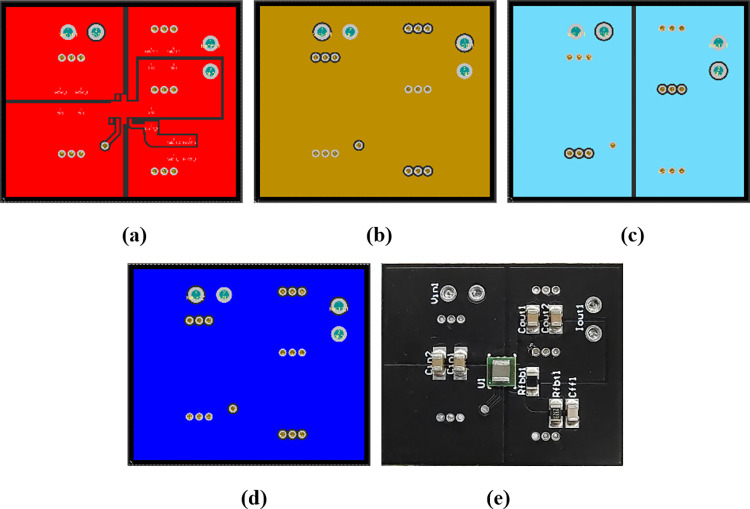
Layouts of the PCB. (a) first layer. (b) second layer. (c) third layer. (d) fourth layer. (e) top surface photo of the PCB.

A four-layer PCB for generating DC 3.3V was also modeled using the test solver. To assess the modeling accuracy, the board was also thermally imaged under natural convection (still air) and radiation heat transfer conditions. The circuit schematic and PCB layouts are illustrated in Figs 14 and 15, respectively.

To achieve a compact circuit, LMZ20501SIL was used as the control IC, in which an inductor is integrated. The initial circuit schematic and PCB layouts were generated using TI’s WEBENCH POWER DESIGNER, and the layouts were then redesigned to obtain a smaller size. The copper planes were nearly fully coated for each layer to improve shielding against external interference, create plate capacitors at both the input and output of the power supply, and enhance heat diffusion.

The board is a standard FR-4 type with surface dimensions of 25 mm x 30 mm. According to the manufacturer’s specifications, each prepreg layer is approximately 0.639 mm thick, and the core layer is 0.15 mm thick. The top and bottom copper foils are 35μm thick, whereas the other two middle copper layers are 30μm thick. Each via wall is approximately 18 μm thick.

### 4.2 Modeling and measurement

In the model built in the test solver, firstly it was necessary to determine the dissipated power of the components. Through simulation using the WEBENCH POWER DESIGNER, it was determined that with a 5V input and 3.3V/1A output, the control IC *U1* resulted in nearly 99.9% of the total heating power loss of all the components. Hence, in the model *U1* was approximated as the only heating component. In the iterations for the analysis of temperature-dependent radiation heat transfer, the temperature distributions were thus also used for electro-thermal Joule heating analysis of metal layers. Electrical analysis was separately conducted for the input (*V*_*in*_) traces, output (*V*_*out*_) traces and ground (*GND*) traces based on the connections between layers.

The thermal resistance between a component’s junction and each pad cell, *R*_*pc*_, is approximately considered equal to each other. *R*_*θJB*_ represents the average thermal resistance between the board and a component’s junction, and is more appropriate than *R*_*θJC(bot)*_ for indicating the thermal resistance between the component’s pads and its junction. This choice was made because *R*_*θJC(bot)*_ is measured with the thermal conductive glue filled under the components [28], and using this parameter in the model would lead to an overestimation of heat conduction between the components and board. Therefore, *R*_*pc*_ can be approximately derived based on *R*_*θJB*_, and can be used to include heat transfer between the component and the board in the model. Additionally, *R*_*θJC(top)*_ can also be used to include heat transfer between the component’s surface and the environment [17]. The thermal-resistance parameters of ceramic capacitors were derived based on an approximate relation [17] between the equivalent thermal conductivity of the capacitor and the capacitance. The thermal-resistance parameters of those resistors were already specified by the manufacturer [29]. Table 2 lists components’ thermal-resistance parameters, radiation areas and emissivities considered in the model.

**Table 2:** Components’ type, *R*_*θJB*_ and *R*_*θJC(top)*_, as well as Area (*A*_*R*_) and Radiation Emissivity(*ℇ*) Considered for Radiation.

Components (Type)	RθJB(°C/W)	RθJC(top)(°C/W)	AR(mm2)/ℇ
U1(LMZ20501SIL)	9.4	20.8	12.95/0.9
R_fbt1_(CRCW0805232KFKEA)	38	0	2.52/0.88
R_fbb1_(CRCW080551KIFKEA)	38	0	2.52/0.88
C_out1_(C0805C106K8PACTU)	15.92	0	3.7/0.94
C_out2_(C0805C106K8PACTU)	15.92	0	3.7/0.94
C_in1_(GRM21BR61A226ME44L)	15.31	0	3.33/0.94
C_in2_(GRM21BR61A226ME44L)	15.31	0	3.33/0.94
C_ff1_(C0805C120J5GACTU)	49.31	0	3.33/0.94

**Fig 16:**
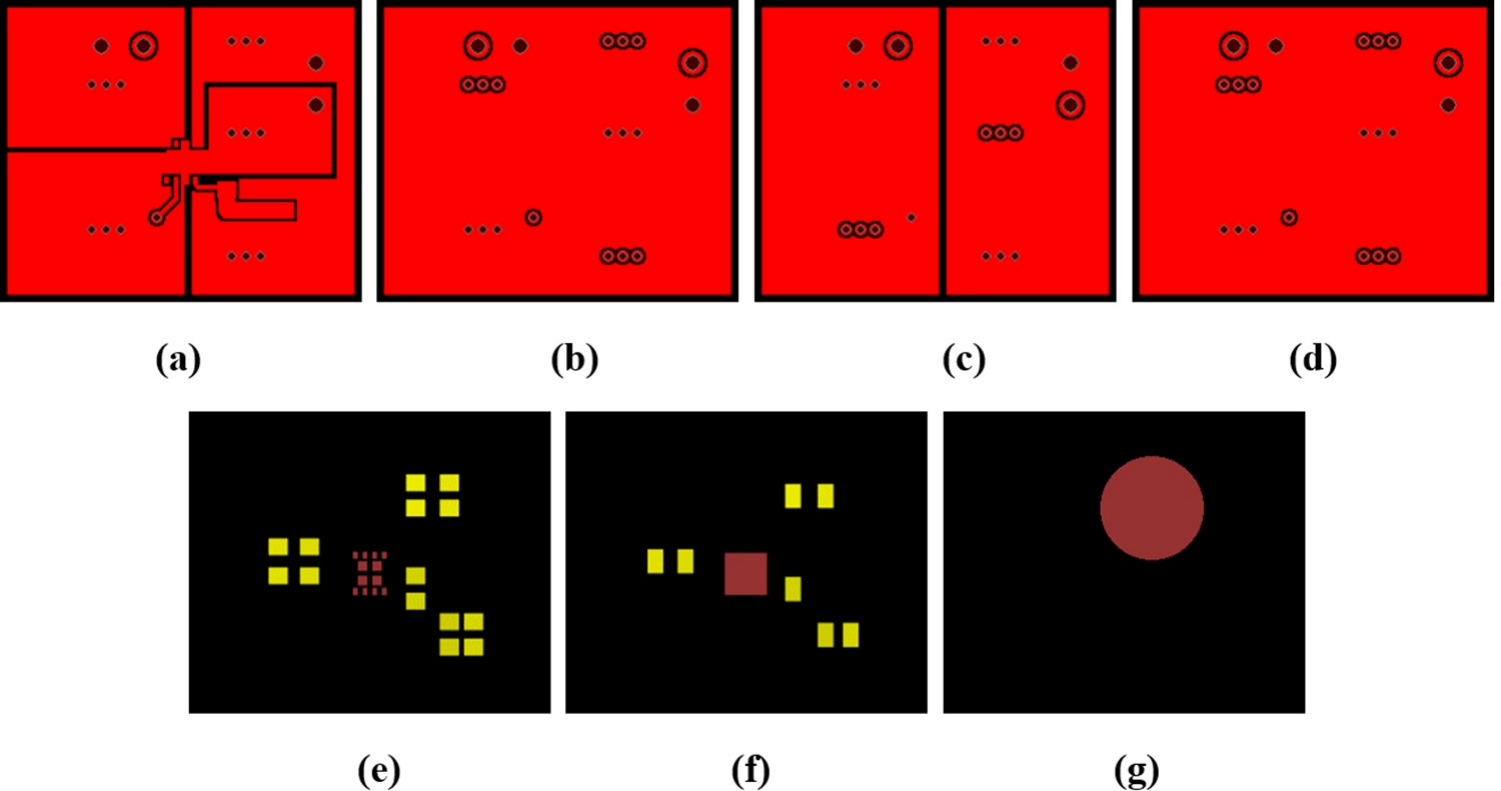
Layout maps of the PCB used for modeling: (a) first layer. (b) second layer. (c) third layer. (d) fourth layer. (e) soldering pads. (f) components’ area. (g) coverage region of the nylon stick.

The layout maps used for modeling have a resolution of 250 x 300 and are illustrated in Fig 16. Hence, the first-level discrete cell had a length of 0.1 mm. The emissivities of the PCB surface and IC were set to 0.9 [2,30,31], except for the regions of the vias and soldering, whereas the emissivity of ceramic capacitors was set to 0.94 [31] and that of resistors was set to 0.88 [31]. The environment was considered a blackbody. The average HTC of all the sides of the stack was set to 10 W/m^2^K, corresponding to the boundary condition of natural convection (still air) [1]. As previously mentioned, the thermal conductivity of the insulating layers was set to 0.3 W/mK, and that of the copper layers was set to 316 W/mK [26]. The ambient temperature was set to 10.2°C based on the measurement.

**Fig 17:**
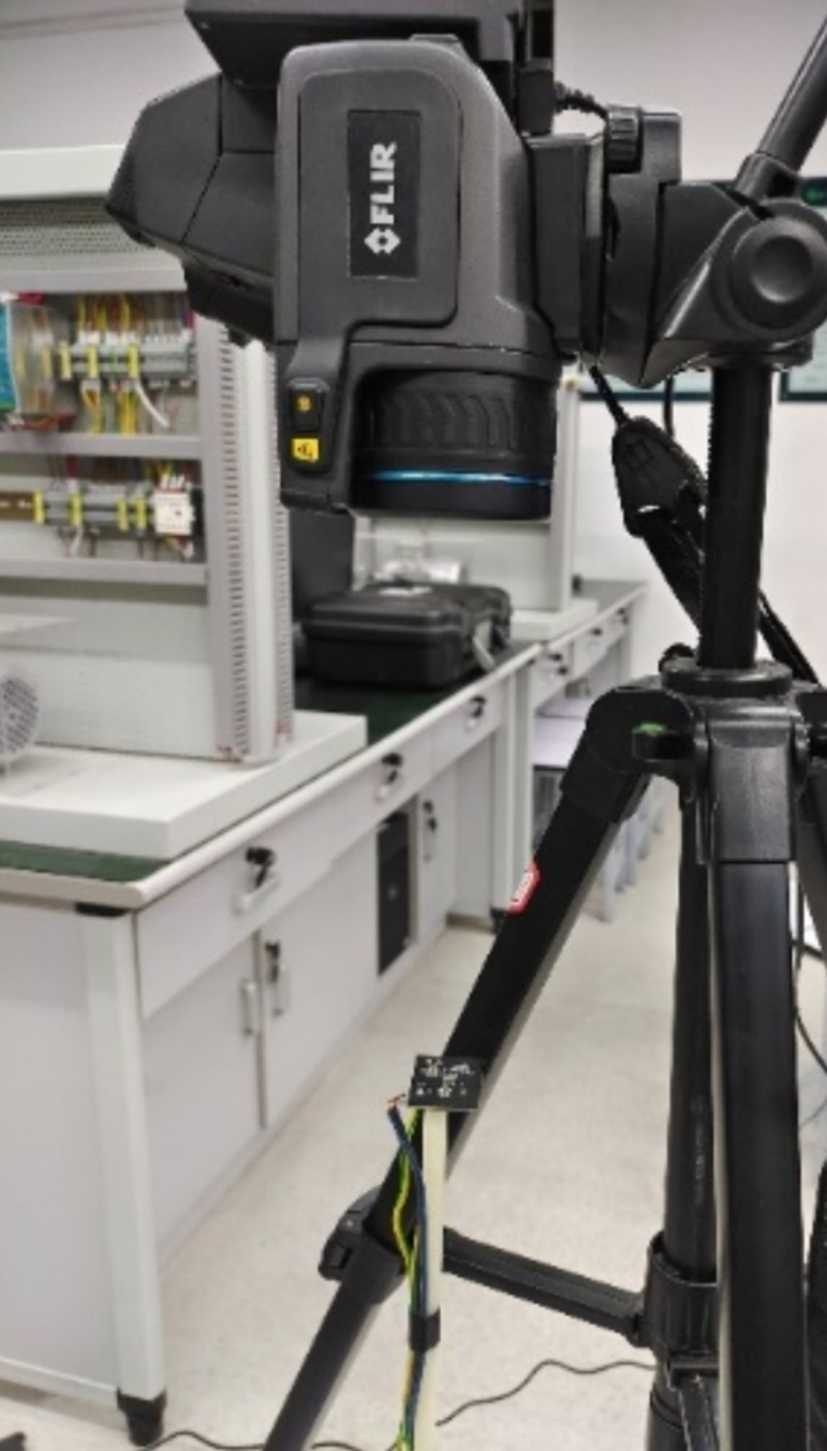
Experimental setup showing the thermal camera and board.

A FLIR T640 thermal camera, as shown in Fig 17, was used to record the temperature. The board was supported by a nylon stick that was about one meter long and 8.6mm in diameter. Heat transfer through the region attached to the nylon stick was neglected. The 5V input was supplied by a programmable DC power supply of RIGOL DP832, and the input current was also measured by the power supply. The output current was sampled by a 100 mΩ (measured resistance: 106 mΩ) resistor and measured by a GDS-2202E oscilloscope. Since there were losses through the lines, the input and output voltages were directly measured at the corresponding terminals of the board. Several cement resistors were used as the load.

**Fig 18:**
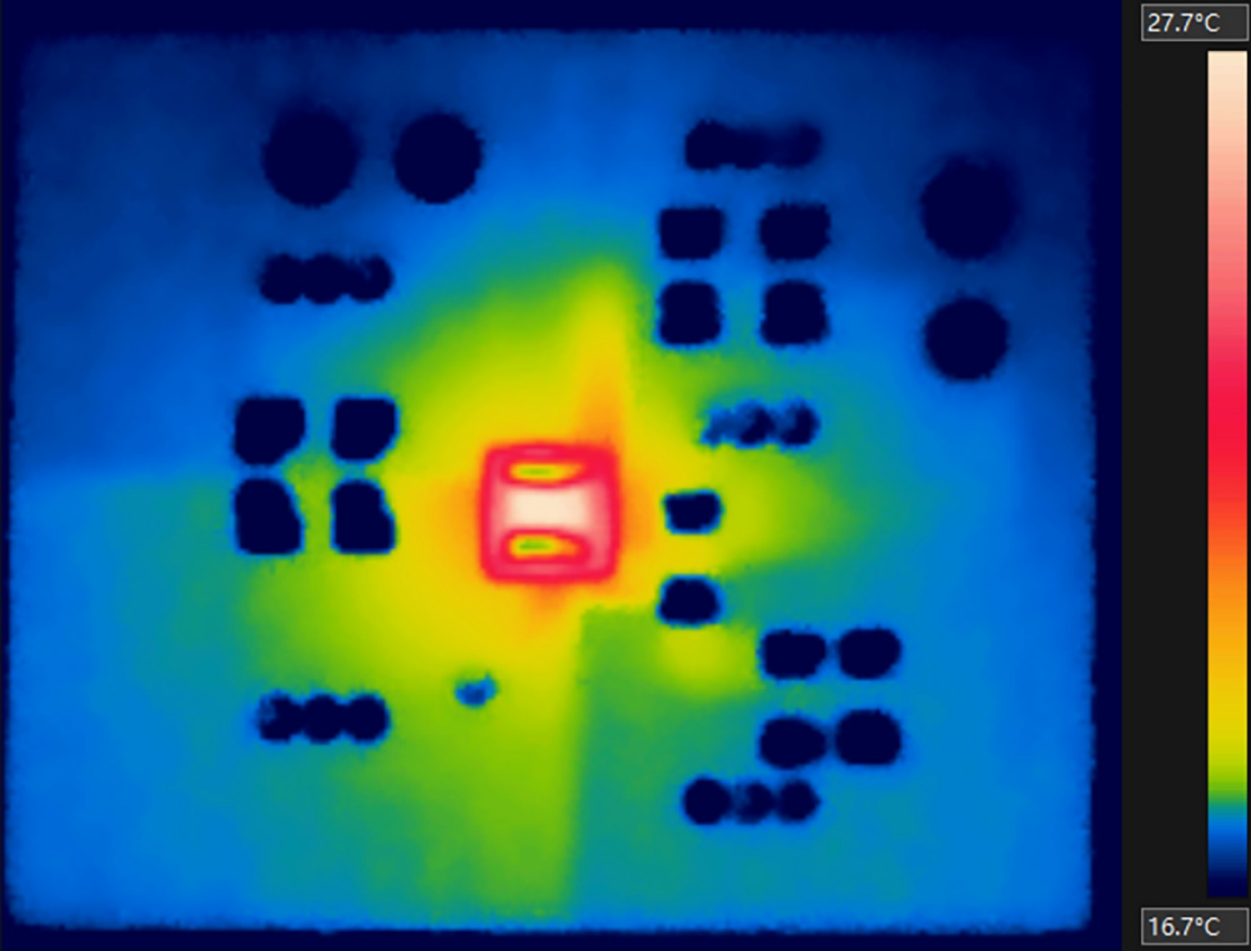
Thermal image of the PCB with Load A.

Initially, the experiment was conducted with a load of about 1A (Load A). After about half an hour from the start, the board reached thermal equilibrium. The average output was about 0.959A and 3.302V, while the input was approximately 4.902V and 0.69A. Hence, the total dissipating power in the PCB (*P*_*td*_) was about 0.216W. Fig 18 shows the thermal image with the top-side temperature of *U1* (*T*_*U1*,*Top*_) at 27.7°C.

**Fig 19:**
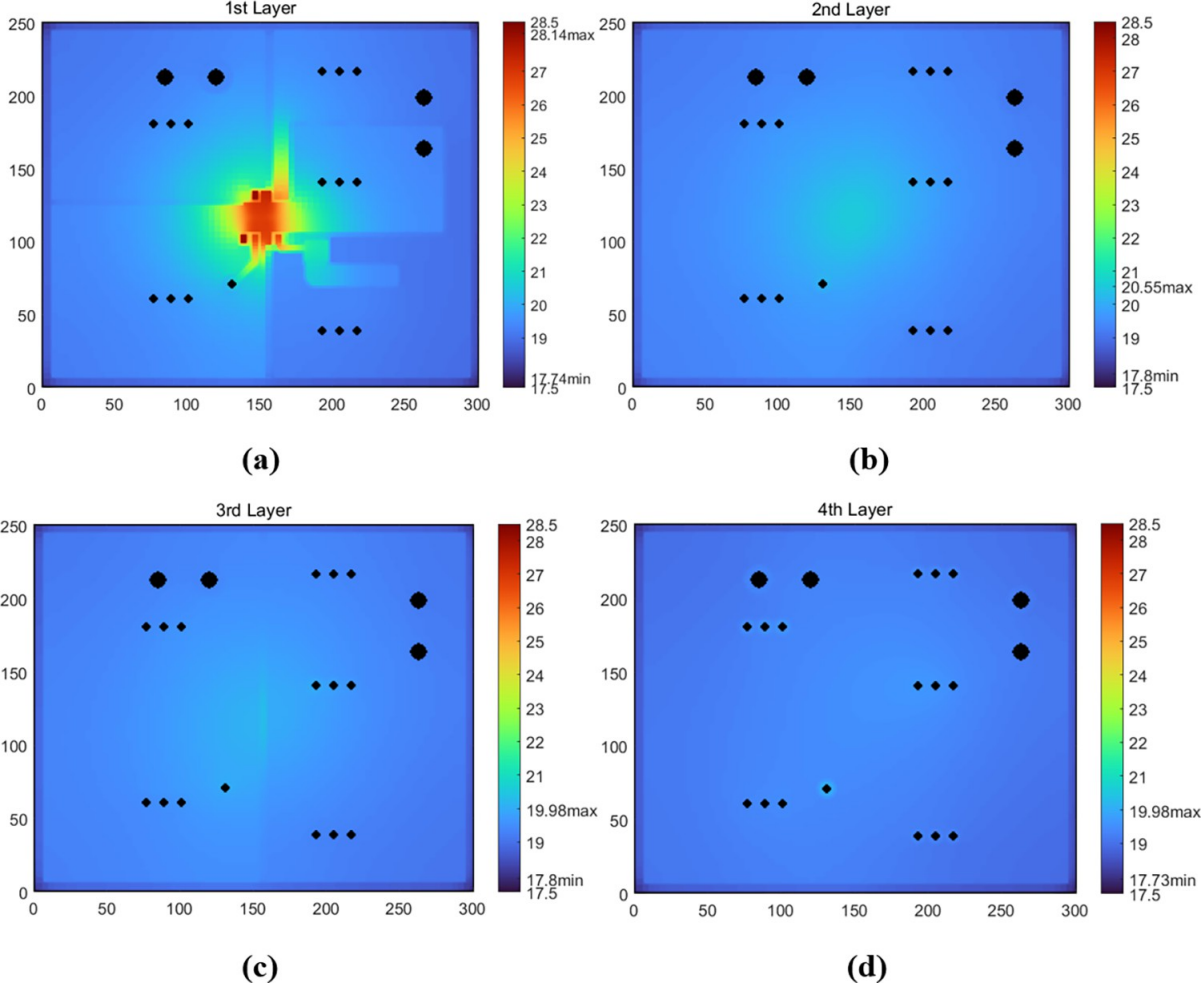
Simulated temperature maps of each layer surface of the PCB with Load A. (a) the first-layer surface. (b) the second-layer surface. (c) the third-layer surface. (d) the fourth-layer surface.

Fig 19 shows the temperature maps of four layer surfaces obtained from the TS model of the PCB. Based on the thermal parameters of *U1*, *T*_*U1*,*Top*_ was further derived to be about 28.48°C, overestimated by about 0.78°C, so that the simulation error rate of *T*_*U1*,*Top*_ was about 4.46%. Moreover, the total Joule heating loss in the circuit was derived to be about 0.004W, thus the heating rate of *U1* was approximately equal to 0.212W, but only about 0.0036W was dissipated from the topside of *U1*. Because of the iterations for electro-thermal analysis of Joule heating, the operation time of the model was about 2 hours.

**Fig 20:**
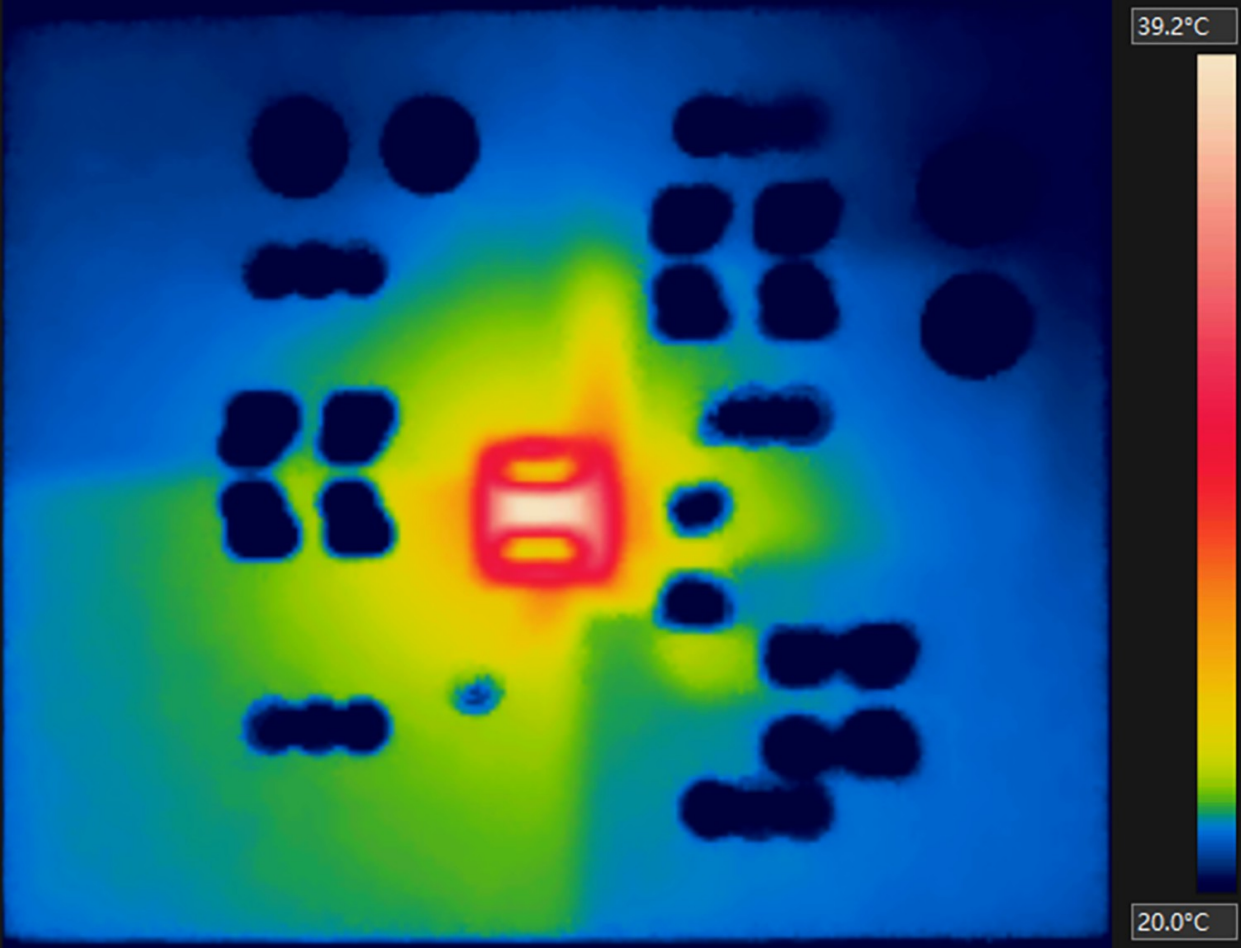
Thermal image of the PCB with Load B.

The experiment was also conducted with a little heavier load (Load B). In the thermal steady state, the average output was about 1.384A and 3.310V, while the input current and voltage were approximately 1.01A and 4.892V. Hence, *P*_*td*_ was about 0.36W. Fig 20 shows the measured thermal image with *T*_*U1*,*Top*_ at 39.2°C.

**Fig 21:**
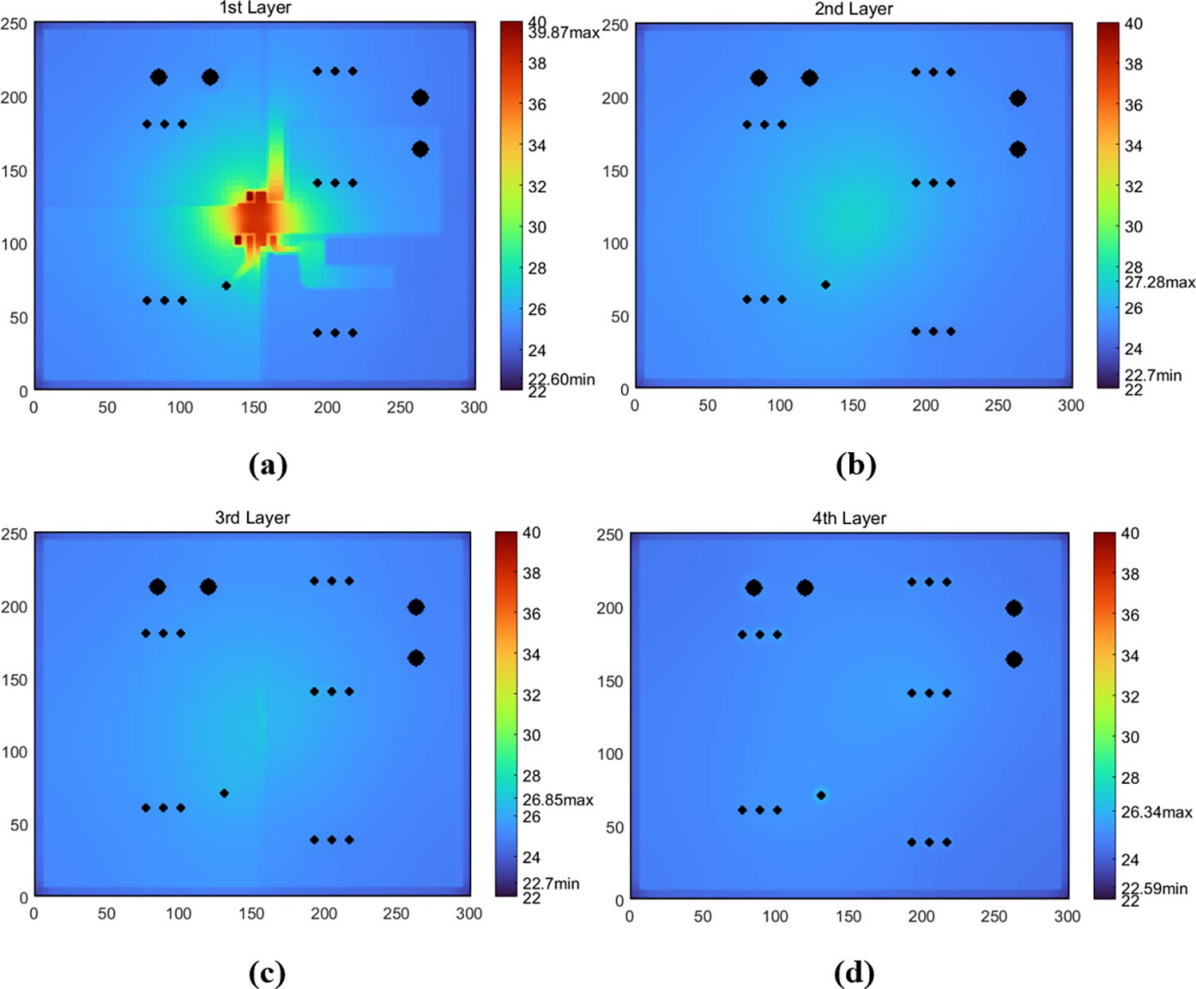
Simulated temperature maps of each layer surface of the PCB with Load B. (a) the first-layer surface. (b) the second-layer surface. (c) the third-layer surface. (d) the fourth-layer surface.

Fig 21 illustrates the modeling results of the PCB with Load B. *T*_*U1*,*Top*_ was further derived to be 40.42°C, overestimated by about 1.22°C with an error rate of about 4.21%. Hence, considering the results for both Load A and Load B, the modeling error rate of *T*_*U1*,*Top*_ was between 4% and 5%. Such error rate can be attributed in part to the approximations used in the modeling method and ignorance of thermal conduction through the connecting wires and nylon stick. Furthermore, the actual power loss from the topside of *U1* under Load B was estimated to be only about 0.006W, resulting in an estimated *U1’s* junction temperature of about 39.72°C, only slightly higher than that of the topside. Hence, most heating power of *U1* was flowing downwards to the board and dissipated from the PCB surfaces. The temperature maps of other three layer surfaces also indicated thermal diffusion through the PCB, highlighting the key role of the PCB structure in the heat diffusion process.

### 4.3 Improving the layout

**Fig 22:**
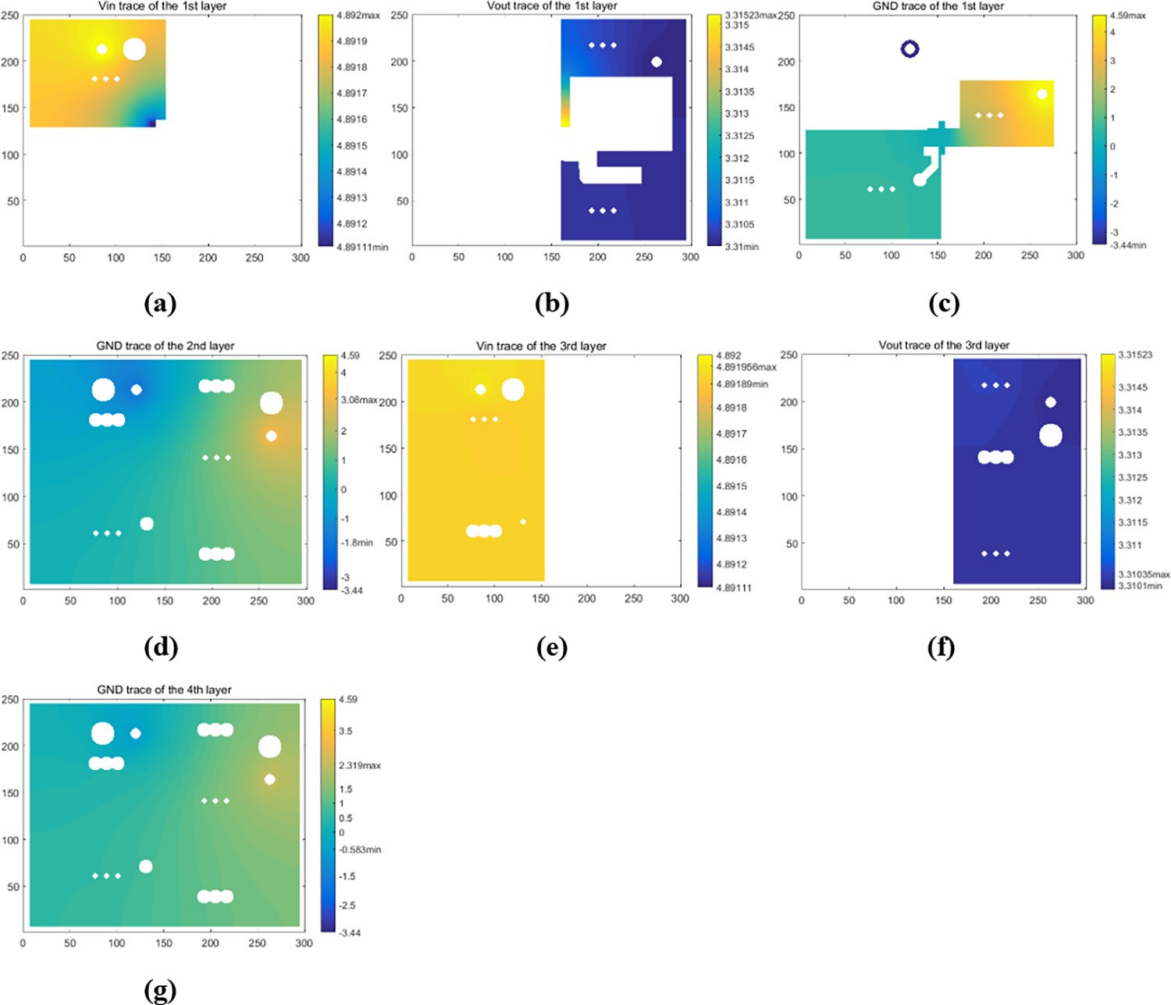
Simulated electric-potential maps of the main-circuit traces of the PCB with Load B. (a) V_in_ (V) trace of the top layer. (b) V_out_ (V) trace of the top layer. (c) V_GND_ (10^-4^V) trace of the top layer. (d) V_GND_ (10^-4^V) trace of the 2^nd^ layer. (e) V_in_ (V) trace of the 3^rd^ layer. (f) V_out_ (V) trace of the 3^rd^ layer. (g) V_GND_ (10^-4^V) trace of the 4^th^ layer.

The electric-potential maps of the PCB traces with Load B are shown in Fig 22. The Joule heating power in *V*_*in*_*/V*_*out*_*/GND* traces and the connected vias was estimated to be 8.79 x 10^−4^ W/7.2x 10^−3^ W/8.86 x 10^−4^ W, respectively. Hence, the total Joule heating loss in traces and vias was about 0.009W, thus the heating rate of *U1* was approximately equal to 0.351W. The IR drop in the traces and vias connected to the input terminals was only 1.1 x 10^−3^ V, but that corresponding to the output terminals was about 6.07 x 10^-3^V. As shown in Fig 22b, half of the output drop was attributed to the relatively thin trace connected to the output pin of *U1*.

**Fig 23:**
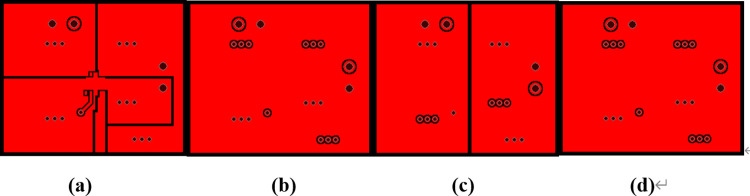
New layout maps of the PCB.

**Fig 24:**
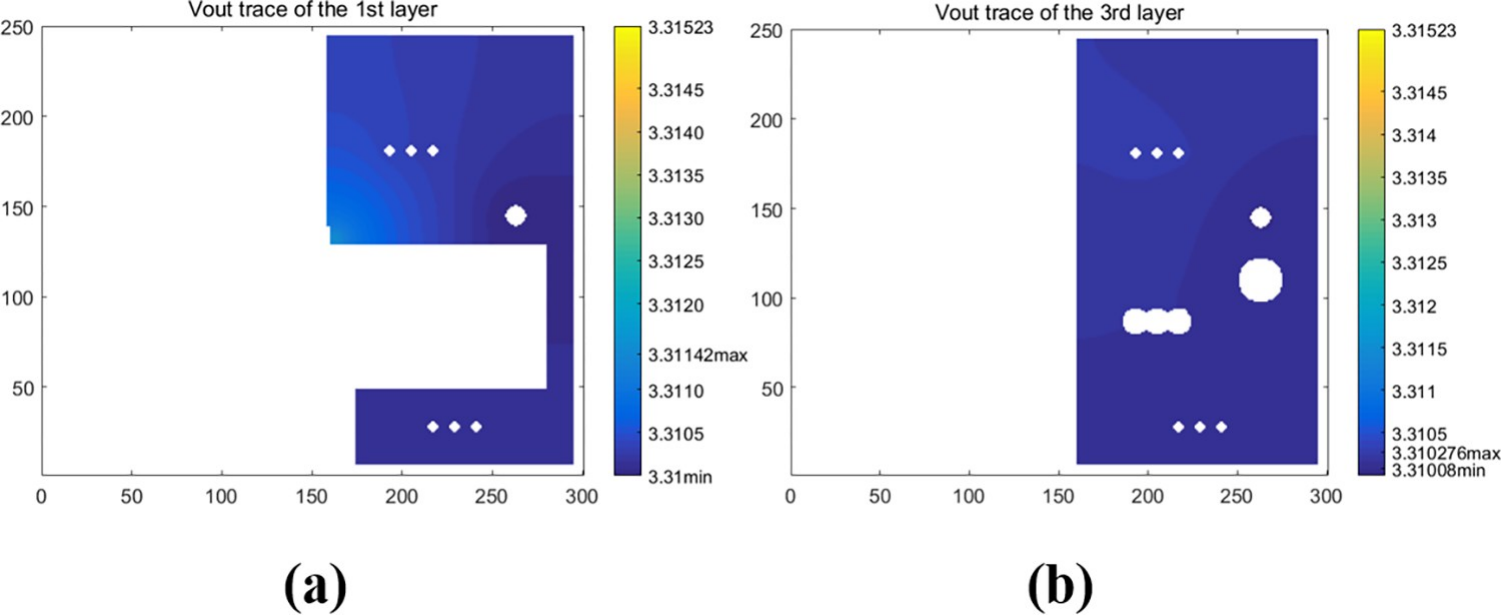
Simulated electric-potential (V) maps of the V_out_ traces of the new layout of the PCB with Load B. (a) the top layer. (b) the 3^rd^ layer.

**Fig 25:**
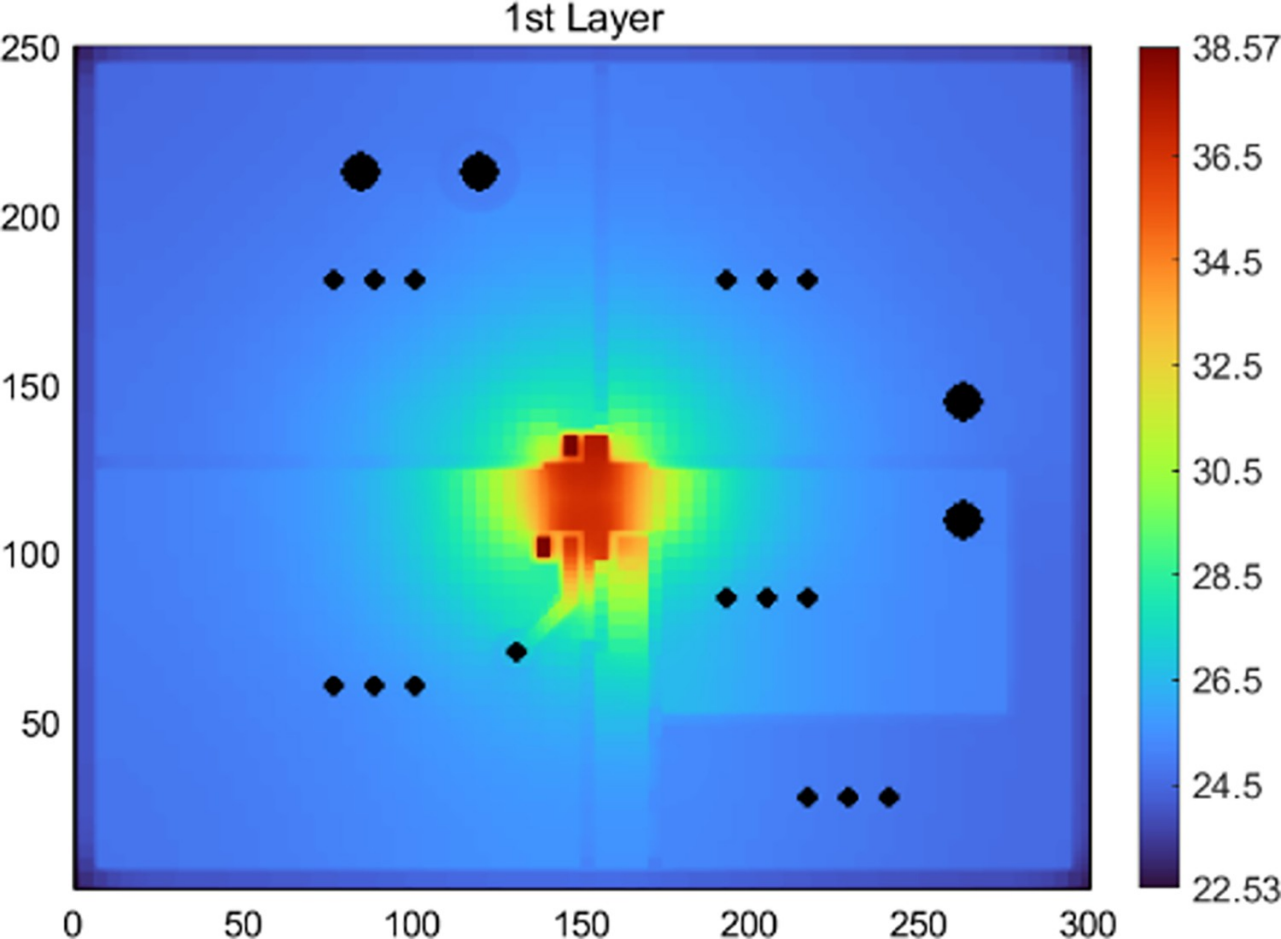
Simulated temperature map of the top layer surface of the new layout of the PCB with Load B.

Hence, as shown in Fig 23, the layout maps were further modified to reduce Joule heating in the *V*_*out*_ traces. In the corresponding model built using the test solver, the heating rate of *U1* was set to 0.351W, then iterations including the electro-thermal Joule heating analysis were running in the same way like the previous model. Finally, the IR drop of the *V*_*out*_ trace in the top layer has decreased to 1.42 x 10^−3^ V as shown in Fig 24, and Joule heating in *V*_*out*_ traces and corresponding vias decreased to 1.9 x 10^−3^ W, which is more than 70% less. Additionally, as shown in Fig 25, the hotspot temperature of the first layer surface has also decreased to 38.57°C, about 1.3°C less. Hence, heat spreading through the traces has also been improved.

## 5. Conclusions

Based on the modeling and experiment results shown in previous sections, the proposed method was verified to be feasible. For the model of a four-layer structure, the average modeling error rate using the test solver was less than 3% based on comparison with COMSOL Multiphysics, while the corresponding running time was less than 9 minutes, much lower than that of the COMSOL models. Hence, utilizing the analytical solution permits a moderate degree of discretization. For the PCB model, the modeling error rate of the surface temperature of the hottest IC was between 4% and 5%. Therefore, both the “thermally-thick” approximation of the core layer and the numerical approximation of heat transfer through the lateral sides of the PCB were testified to be reasonable. It can be predicted that the method may also be suitable for other multilayer PCB structures with six or more metal-foil layers, as well as for some semiconductor power modules with a laminate structure comprising multiple layers.

## Supporting information

S1 Data (ZIP)
