# Supplementary figures and images for: A modeling method for thermal steady-state simulation of the four-layer printed circuit board
Source: PLoS One. 2024 Sep 18;19(9):e0310237. doi: 10.1371/journal.pone.0310237 (PMC11410250; doi:10.1371/journal.pone.0310237)

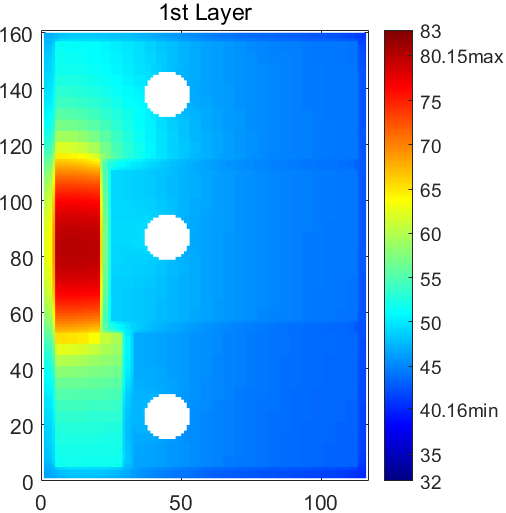

Supplement: S1 Data — (ZIP) [file pone.0310237.s001.zip › new figure data/FIG10-a-NoB015new.png]

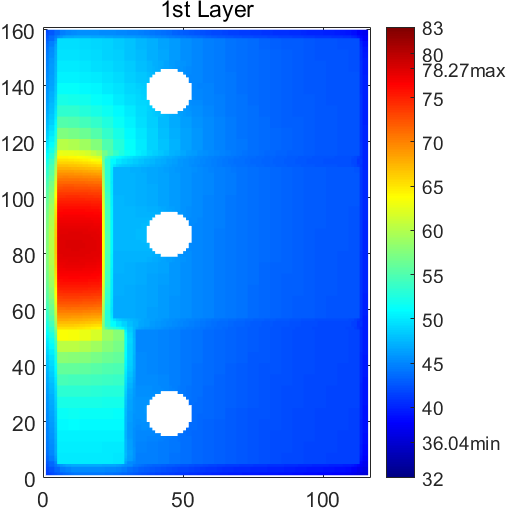

Supplement: S1 Data — (ZIP) [file pone.0310237.s001.zip › new figure data/FIG10-b-HS015new.png]

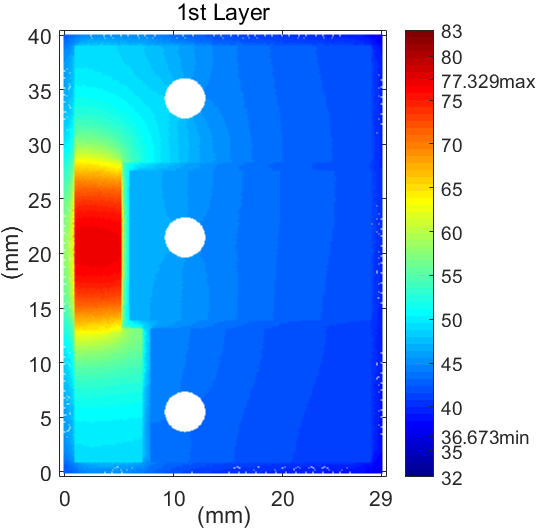

Supplement: S1 Data — (ZIP) [file pone.0310237.s001.zip › new figure data/FIG10-c-COMSOL-L1-015.png]

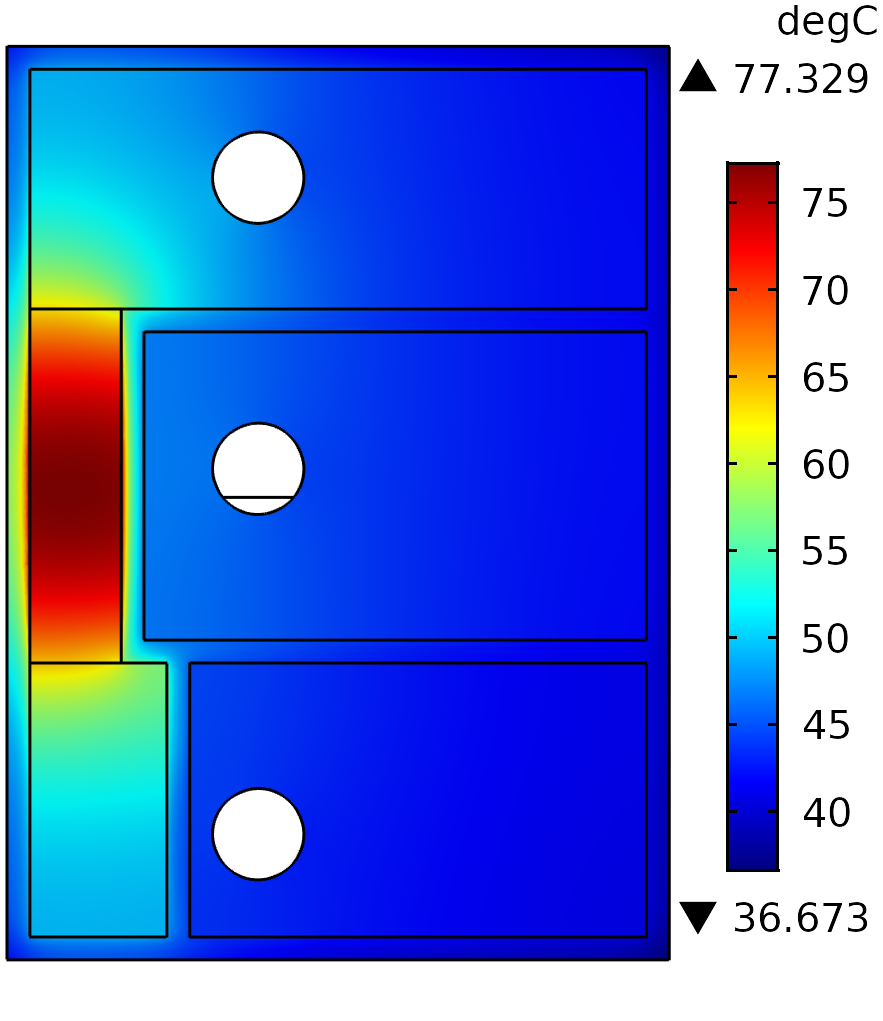

Supplement: S1 Data — (ZIP) [file pone.0310237.s001.zip › new figure data/FIG10-d-L1-015.png]

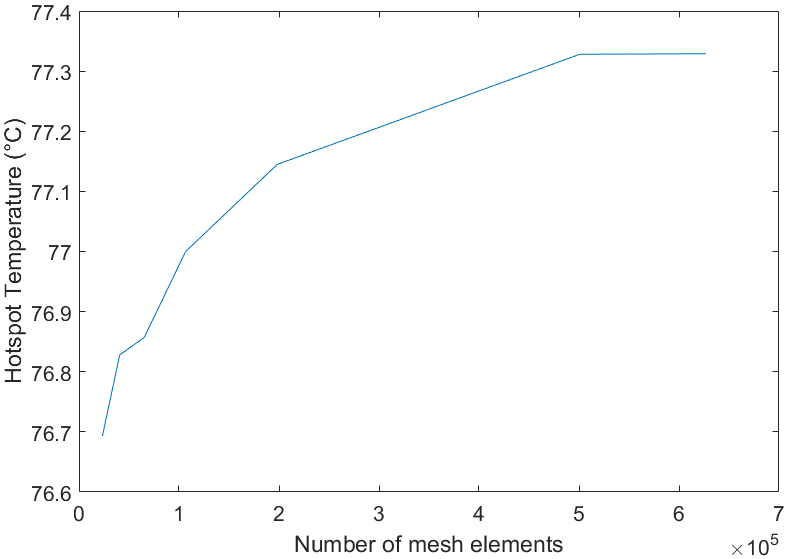

Supplement: S1 Data — (ZIP) [file pone.0310237.s001.zip › new figure data/FIG10-e-converge.png]

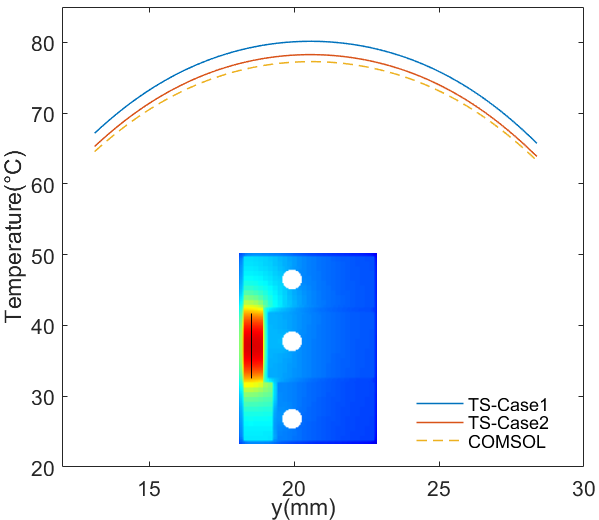

Supplement: S1 Data — (ZIP) [file pone.0310237.s001.zip › new figure data/FIG10-f-3curve015new.png]

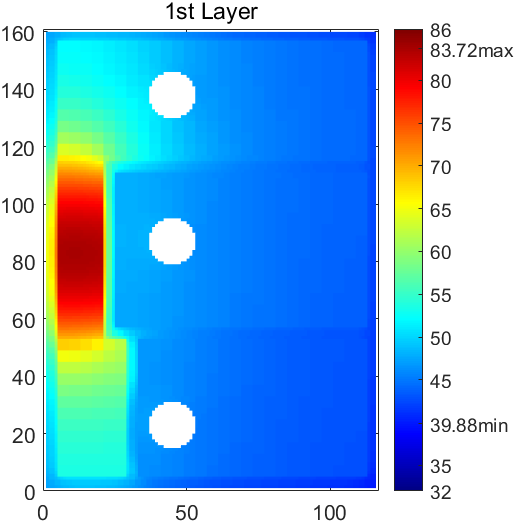

Supplement: S1 Data — (ZIP) [file pone.0310237.s001.zip › new figure data/FIG11-a-NoB055new.png]

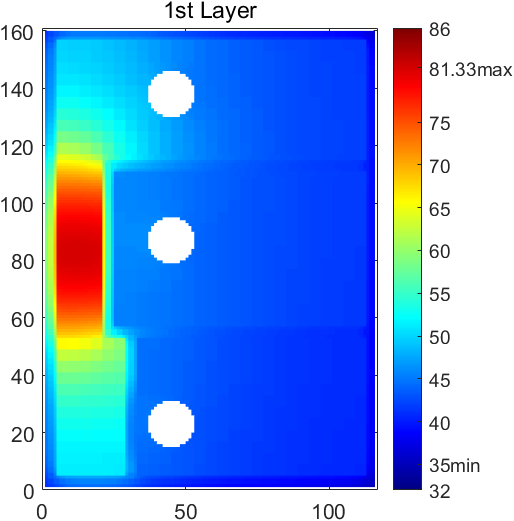

Supplement: S1 Data — (ZIP) [file pone.0310237.s001.zip › new figure data/FIG11-b-HS055new.png]

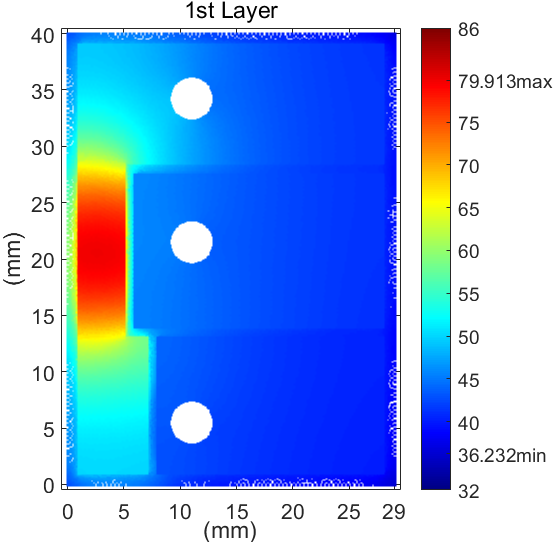

Supplement: S1 Data — (ZIP) [file pone.0310237.s001.zip › new figure data/FIG11-c-COMSOL-055new.png]

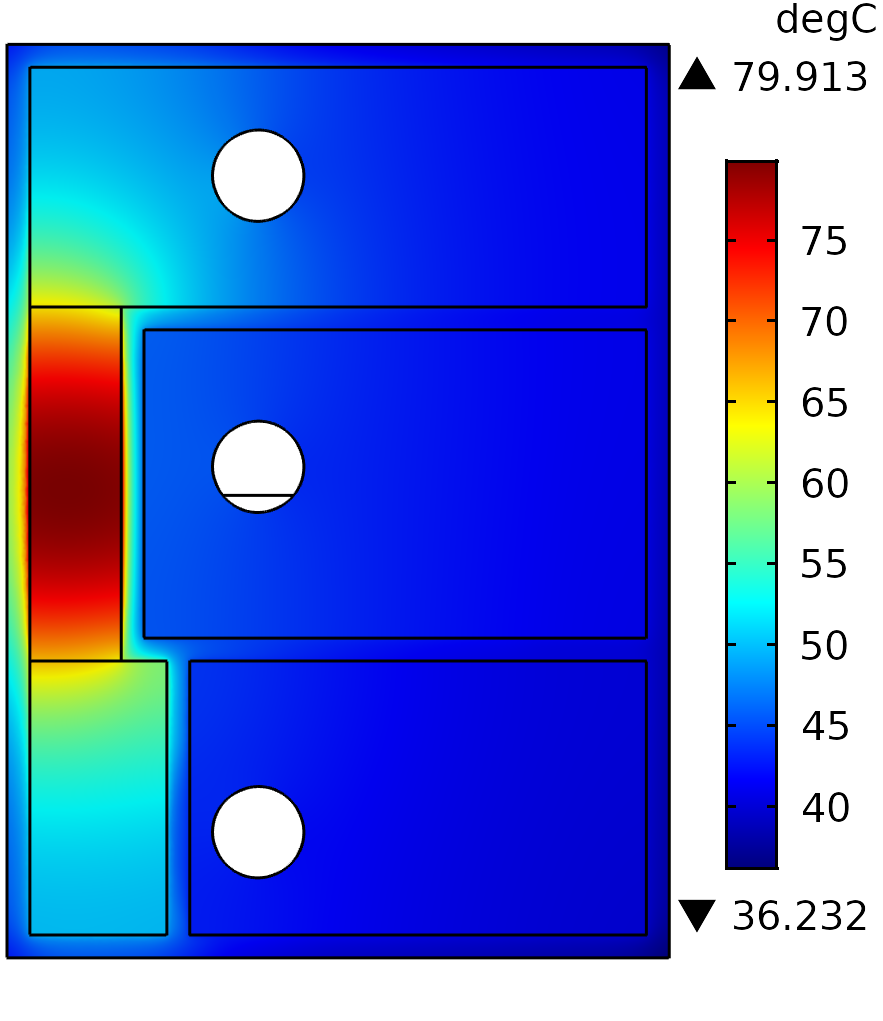

Supplement: S1 Data — (ZIP) [file pone.0310237.s001.zip › new figure data/FIG11-d-L1-055.png]

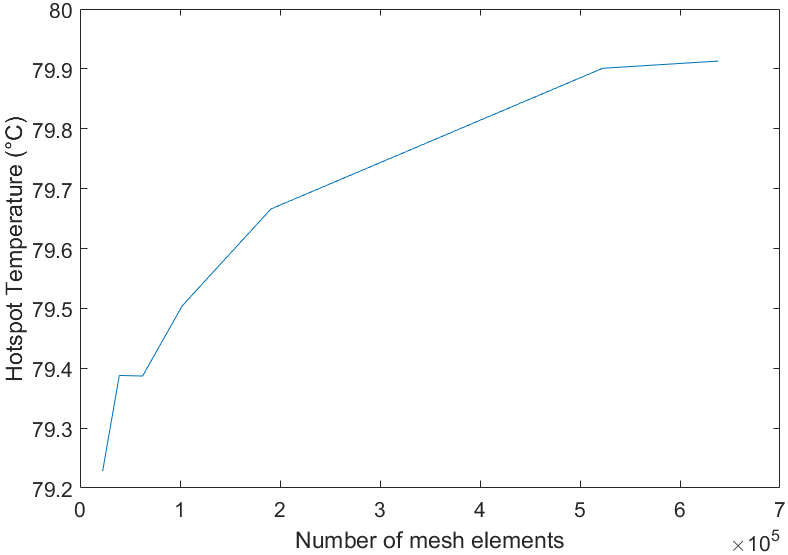

Supplement: S1 Data — (ZIP) [file pone.0310237.s001.zip › new figure data/FIG11-e-converge.png]

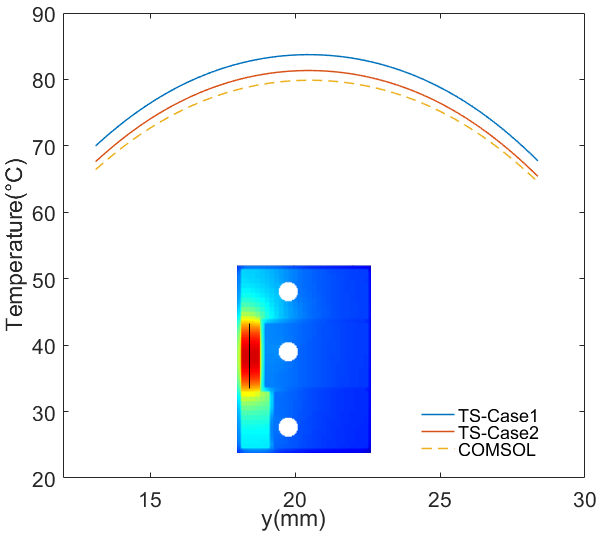

Supplement: S1 Data — (ZIP) [file pone.0310237.s001.zip › new figure data/FIG11-f-3curve055new.png]

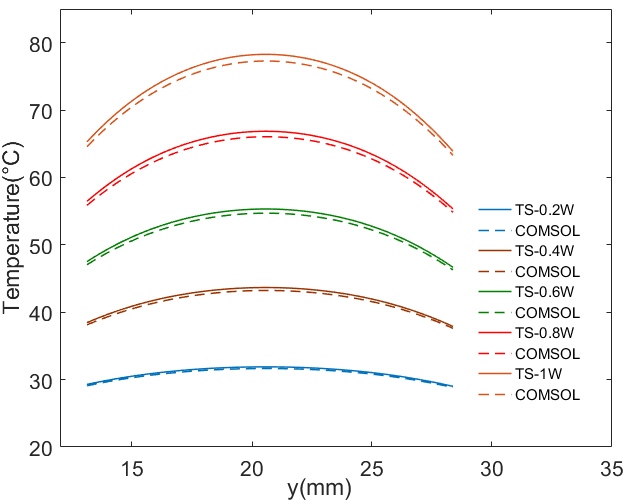

Supplement: S1 Data — (ZIP) [file pone.0310237.s001.zip › new figure data/FIG12-a-015.png]

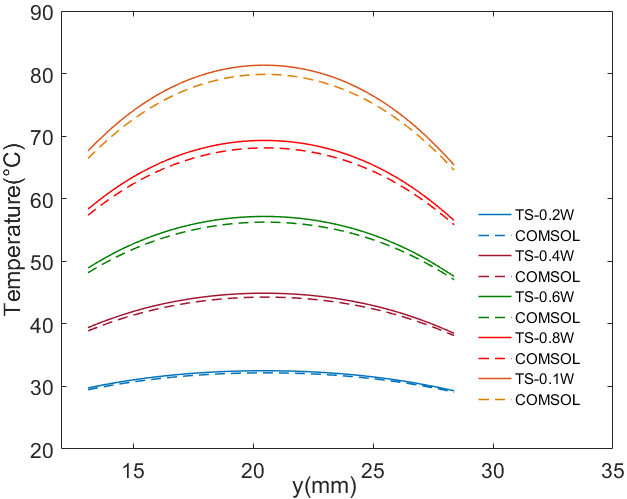

Supplement: S1 Data — (ZIP) [file pone.0310237.s001.zip › new figure data/FIG12-b-055.png]

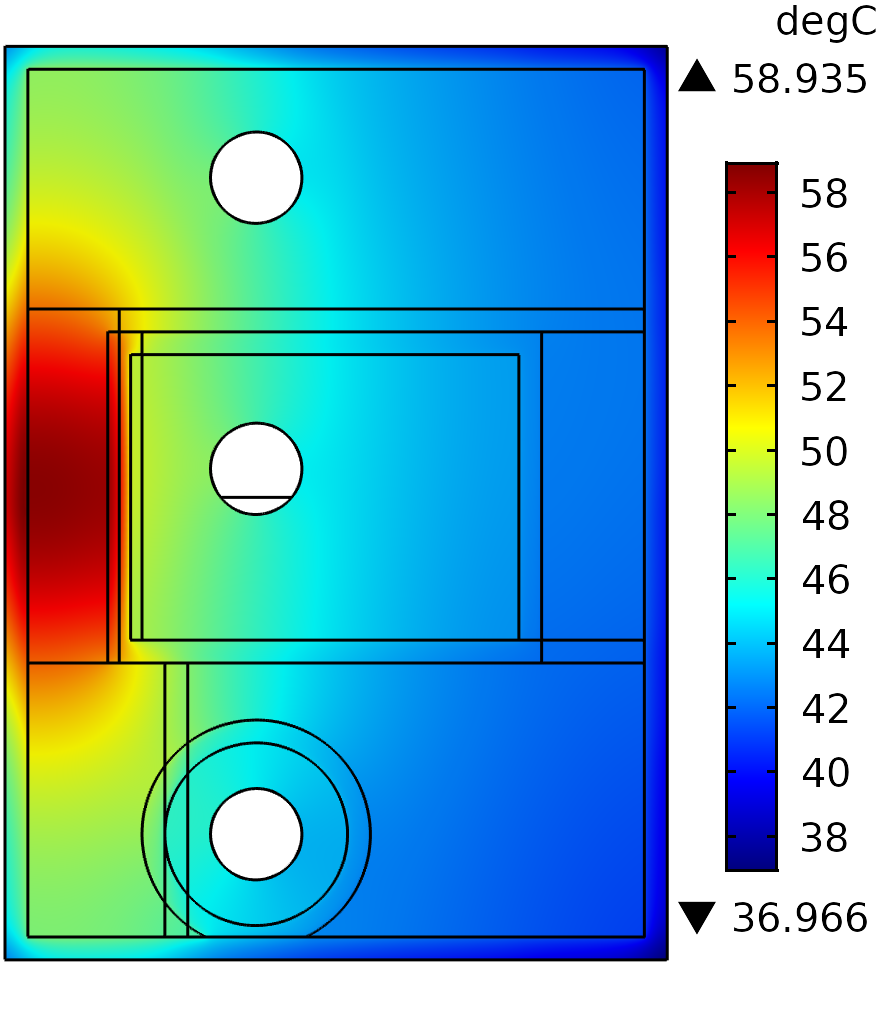

Supplement: S1 Data — (ZIP) [file pone.0310237.s001.zip › new figure data/FIG13-COMSOL-L2-015.png]

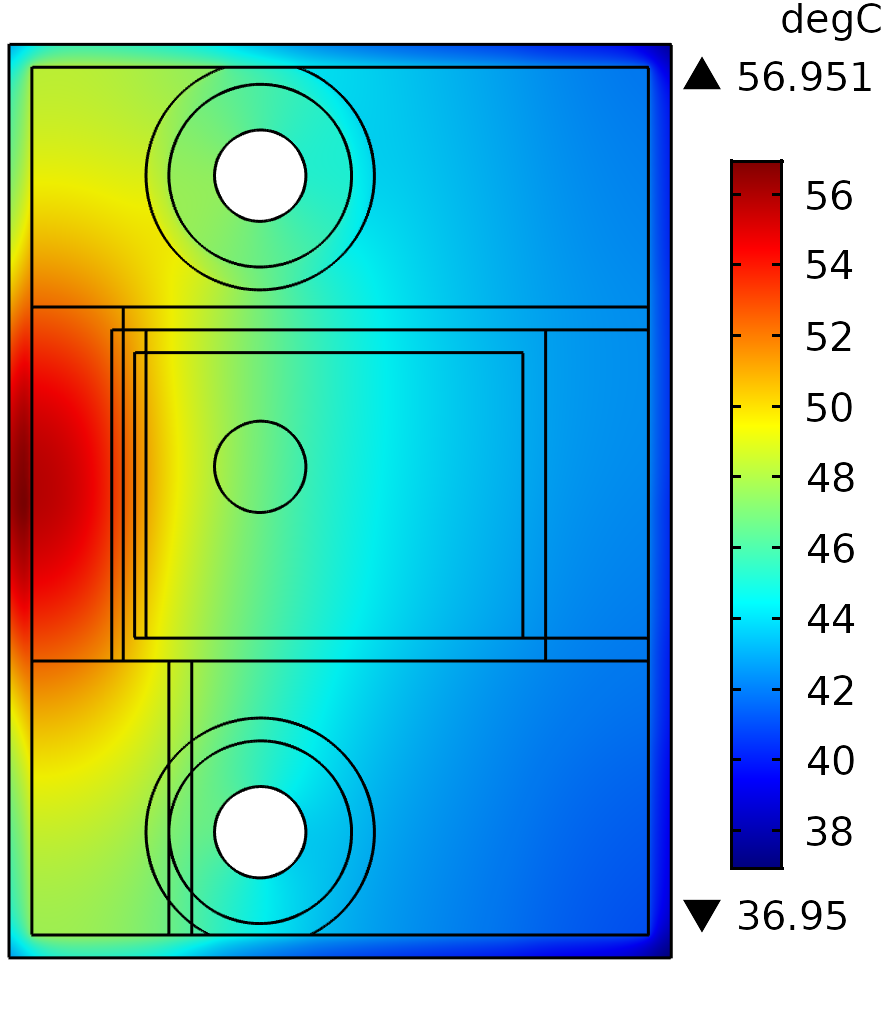

Supplement: S1 Data — (ZIP) [file pone.0310237.s001.zip › new figure data/FIG13-COMSOL-L3-015.png]

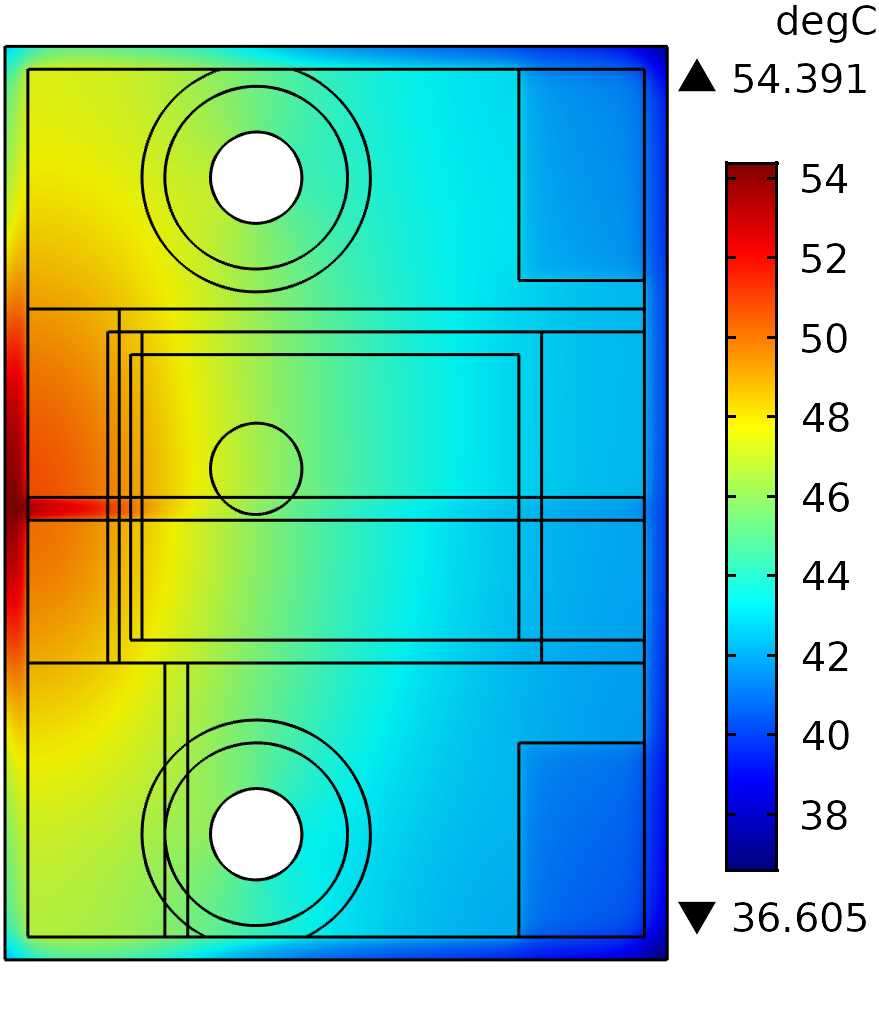

Supplement: S1 Data — (ZIP) [file pone.0310237.s001.zip › new figure data/FIG13-COMSOL-L4-015.png]

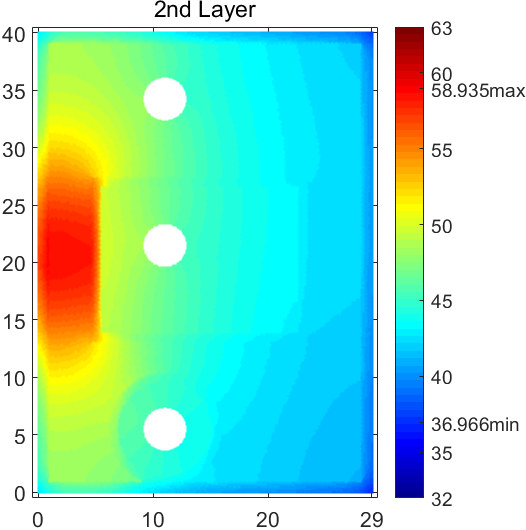

Supplement: S1 Data — (ZIP) [file pone.0310237.s001.zip › new figure data/FIG13-L2-COMSOL-015new.png]

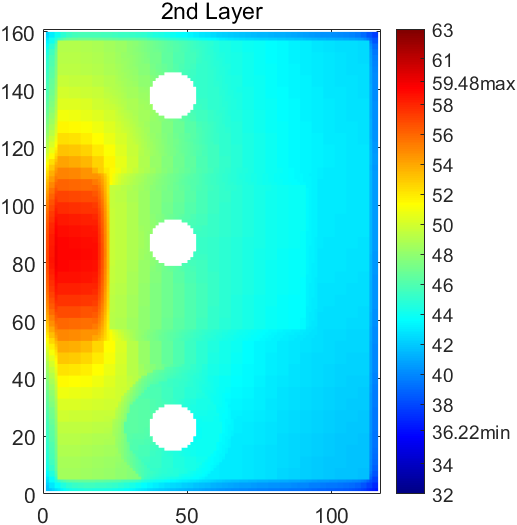

Supplement: S1 Data — (ZIP) [file pone.0310237.s001.zip › new figure data/FIG13-L2-HS015new.png]

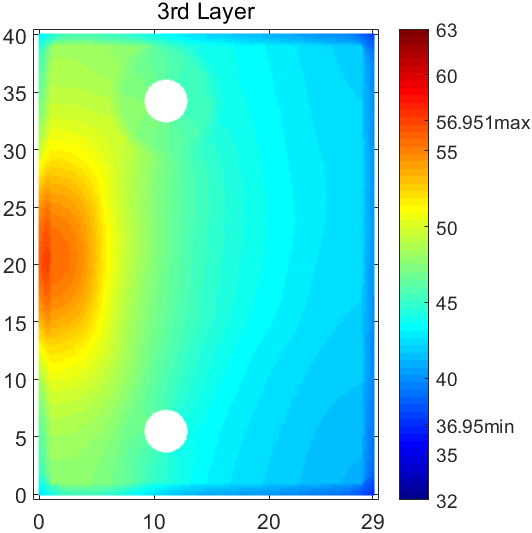

Supplement: S1 Data — (ZIP) [file pone.0310237.s001.zip › new figure data/FIG13-L3-COMSOL-015new.png]

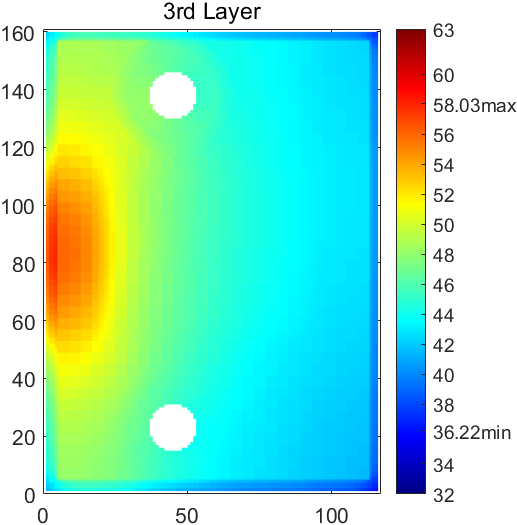

Supplement: S1 Data — (ZIP) [file pone.0310237.s001.zip › new figure data/FIG13-L3-HS015new.png]

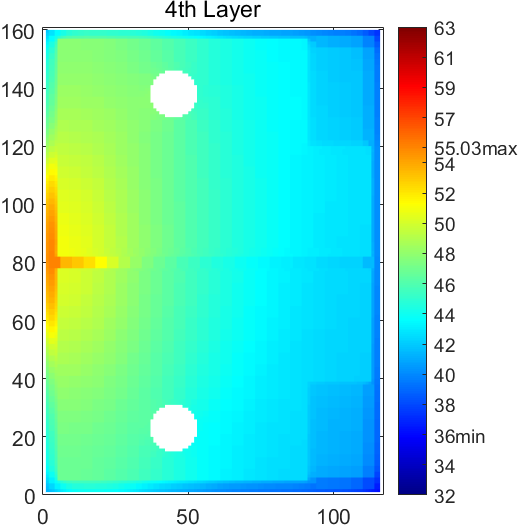

Supplement: S1 Data — (ZIP) [file pone.0310237.s001.zip › new figure data/FIG13-L4-HS015new.png]

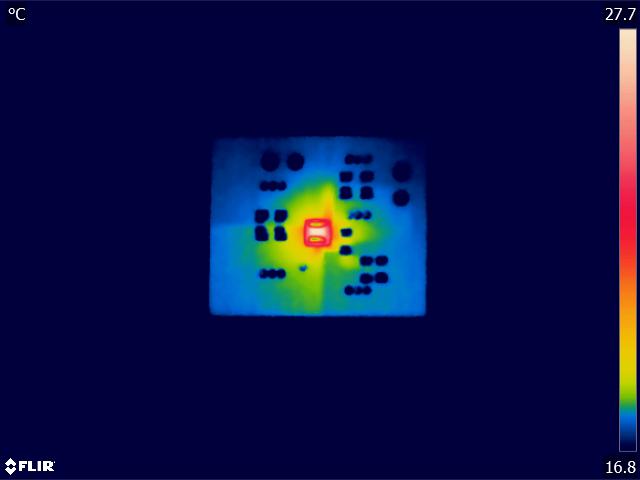

Supplement: S1 Data — (ZIP) [file pone.0310237.s001.zip › new figure data/FIG18-FLIR-277-069.jpg]

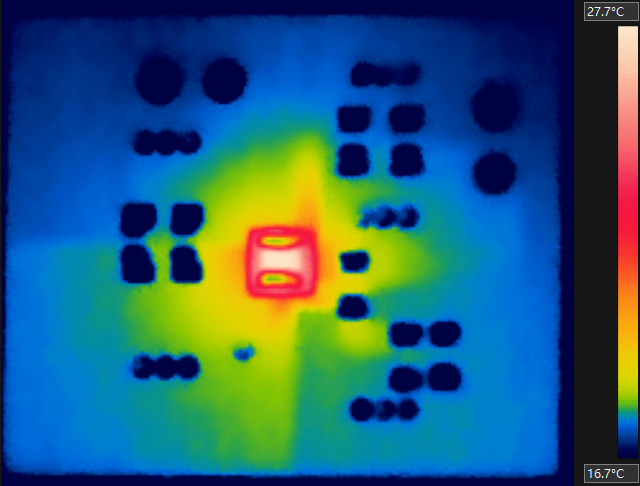

Supplement: S1 Data — (ZIP) [file pone.0310237.s001.zip › new figure data/FIG18-zoom-277.png]

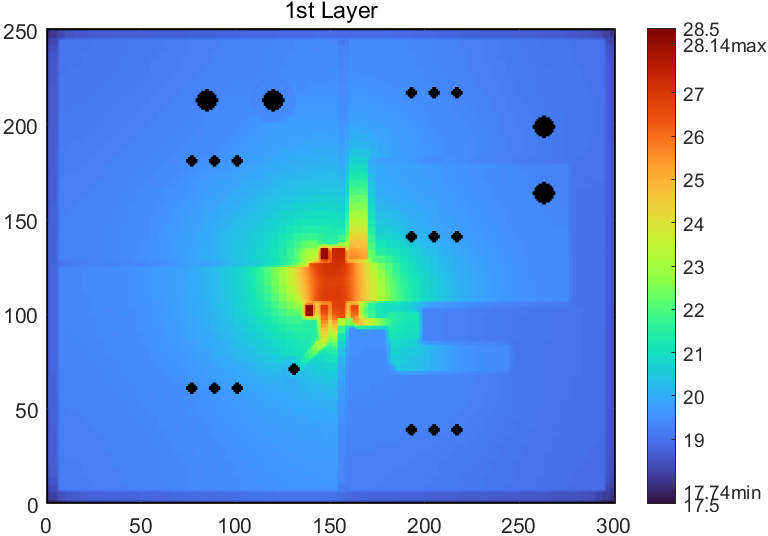

Supplement: S1 Data — (ZIP) [file pone.0310237.s001.zip › new figure data/FIG19-a-277new.png]

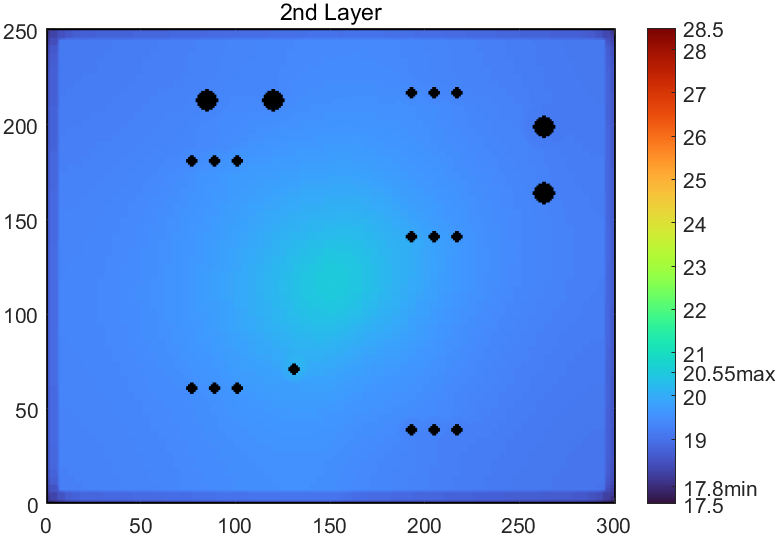

Supplement: S1 Data — (ZIP) [file pone.0310237.s001.zip › new figure data/FIG19-b-277new.png]

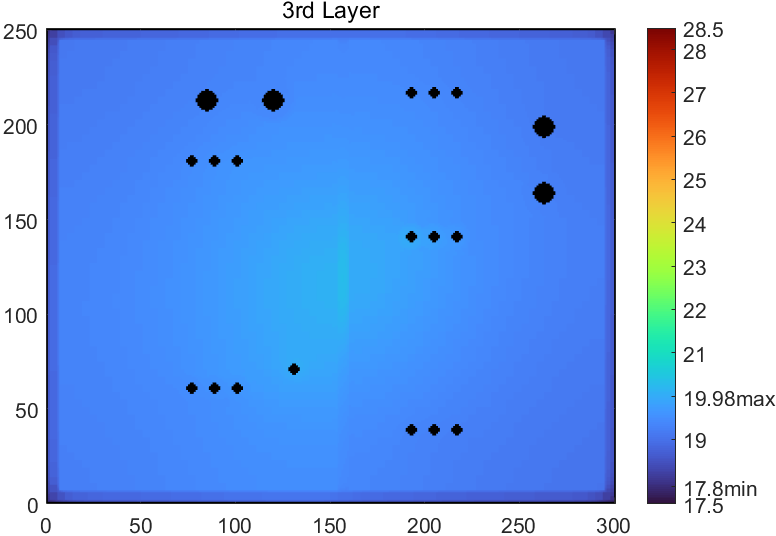

Supplement: S1 Data — (ZIP) [file pone.0310237.s001.zip › new figure data/FIG19-c-277new.png]

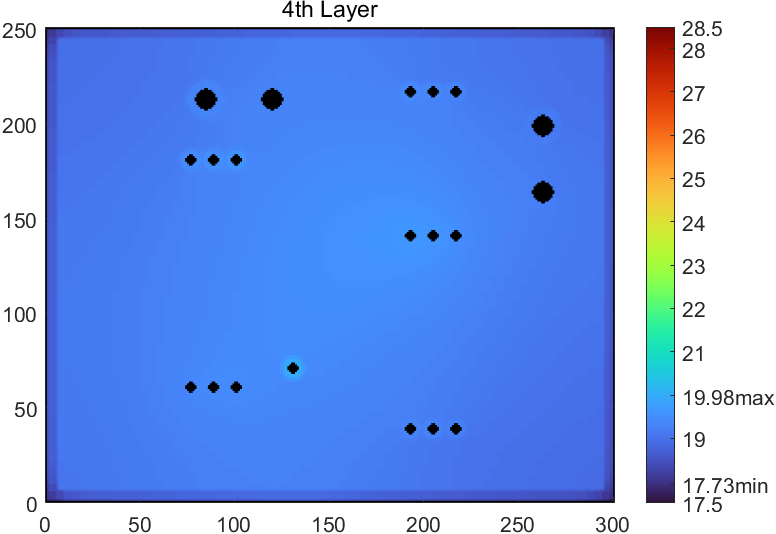

Supplement: S1 Data — (ZIP) [file pone.0310237.s001.zip › new figure data/FIG19-d-277new.png]

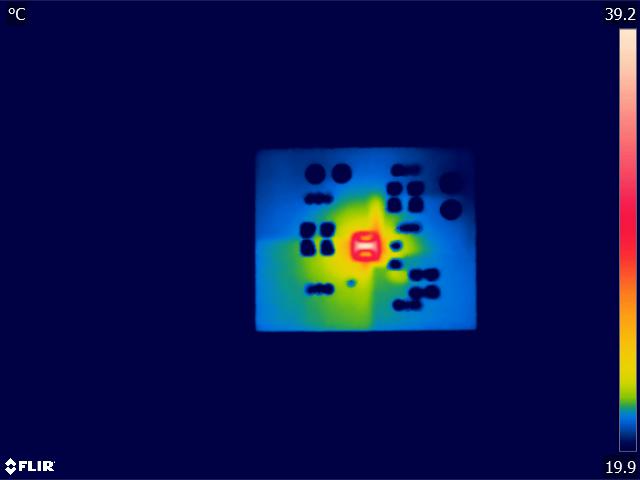

Supplement: S1 Data — (ZIP) [file pone.0310237.s001.zip › new figure data/FIG20-FLIR-392-101.jpg]

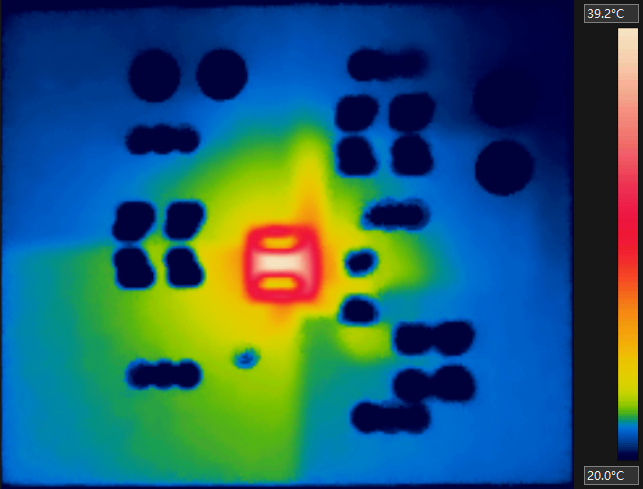

Supplement: S1 Data — (ZIP) [file pone.0310237.s001.zip › new figure data/FIG20-zoom-392.png]

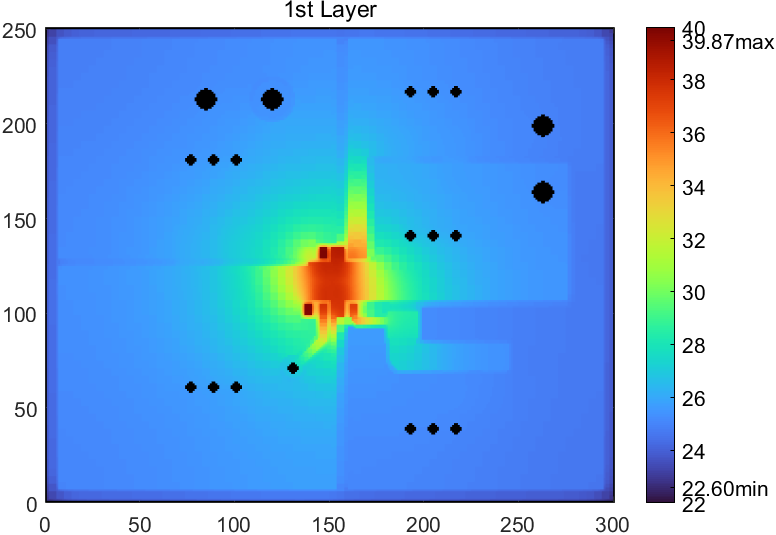

Supplement: S1 Data — (ZIP) [file pone.0310237.s001.zip › new figure data/FIG21-a-L1new.png]

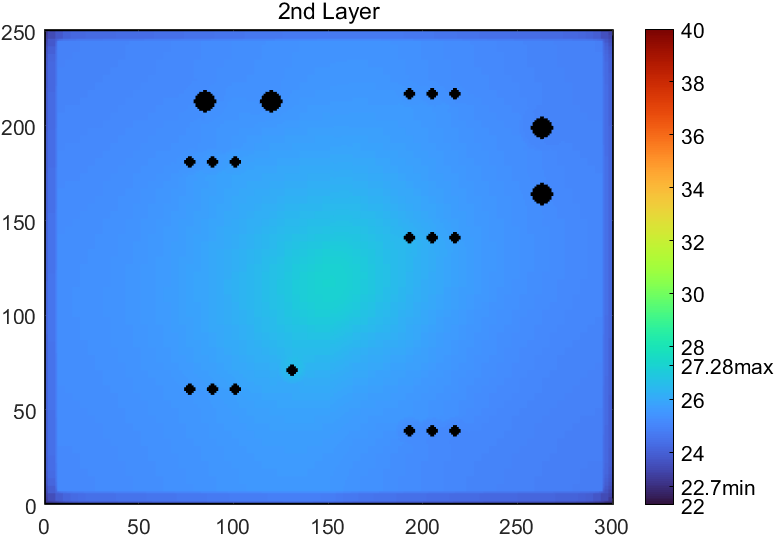

Supplement: S1 Data — (ZIP) [file pone.0310237.s001.zip › new figure data/FIG21-b-L2new.png]

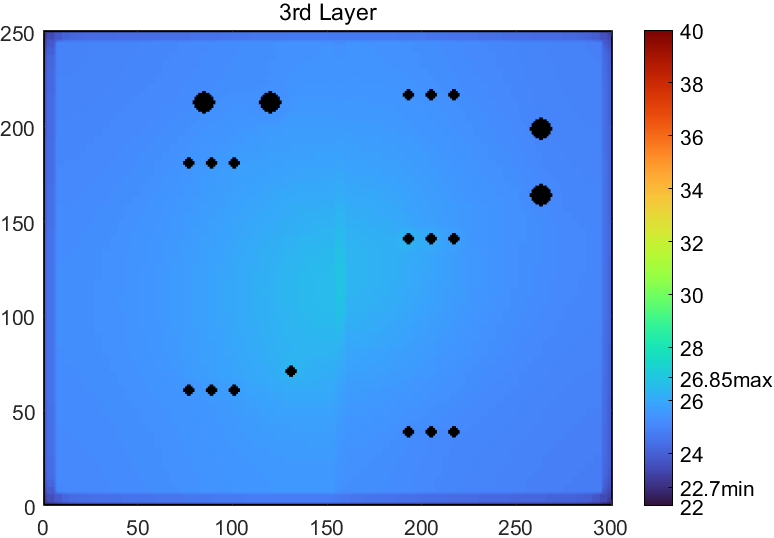

Supplement: S1 Data — (ZIP) [file pone.0310237.s001.zip › new figure data/FIG21-c-L3new.png]

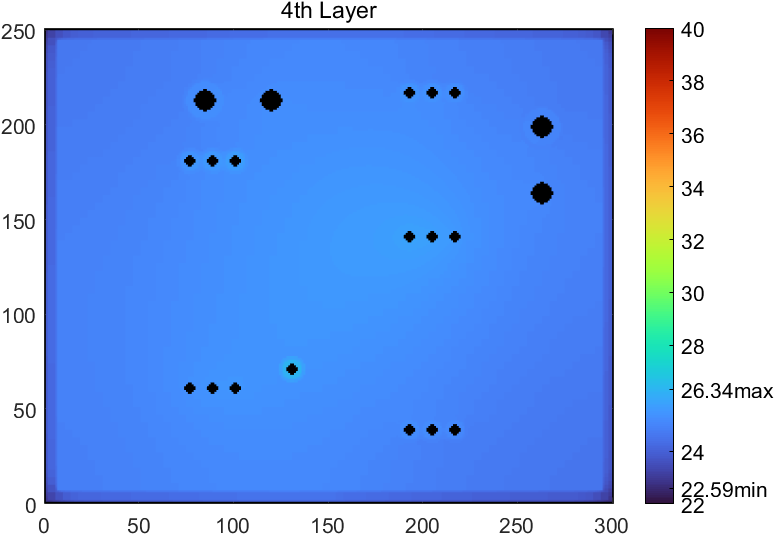

Supplement: S1 Data — (ZIP) [file pone.0310237.s001.zip › new figure data/FIG21-d-L4new.png]

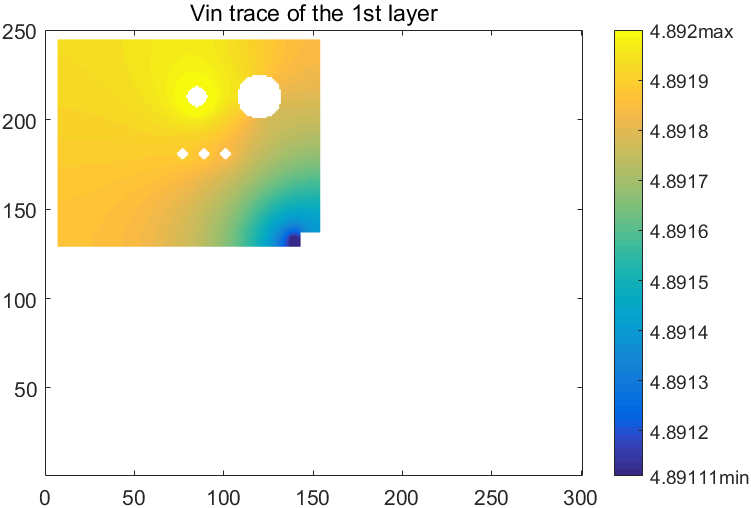

Supplement: S1 Data — (ZIP) [file pone.0310237.s001.zip › new figure data/FIG22-a-VinL1new.png]

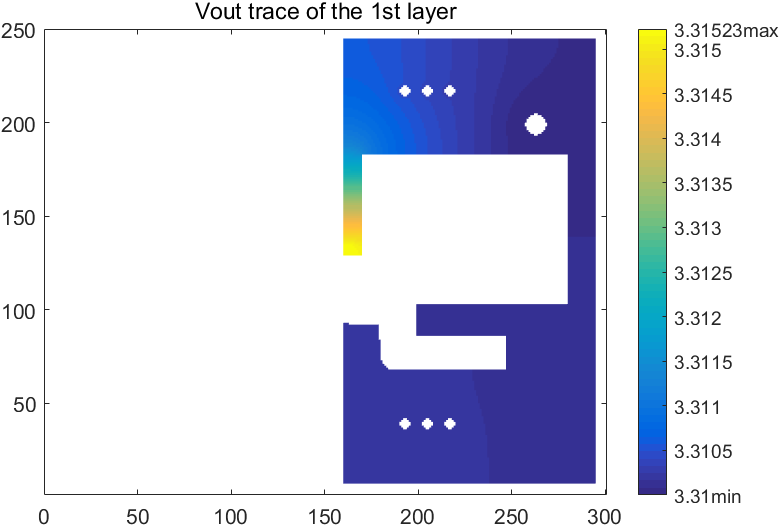

Supplement: S1 Data — (ZIP) [file pone.0310237.s001.zip › new figure data/FIG22-b-VoL1new.png]

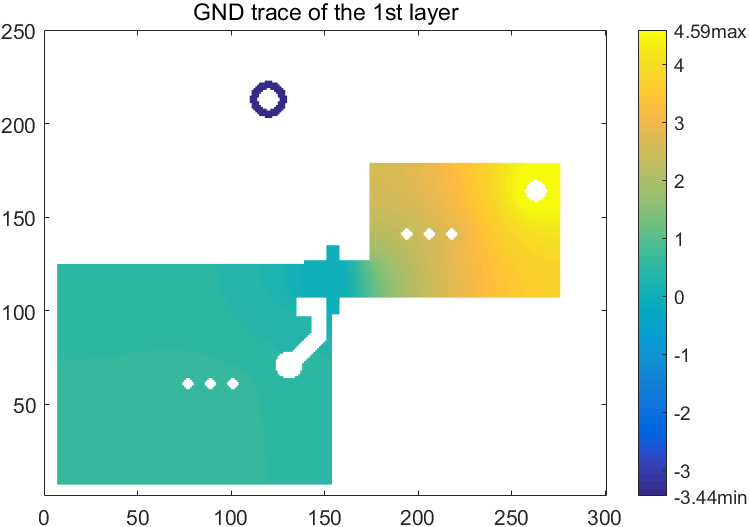

Supplement: S1 Data — (ZIP) [file pone.0310237.s001.zip › new figure data/FIG22-c-GNDL1new.png]

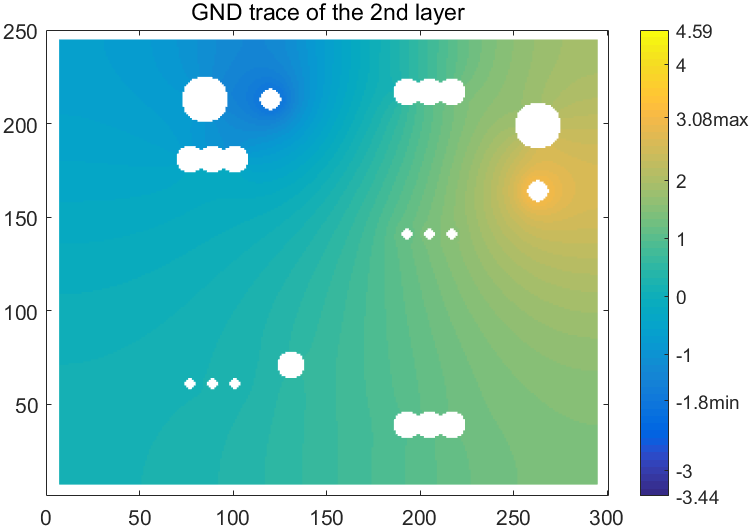

Supplement: S1 Data — (ZIP) [file pone.0310237.s001.zip › new figure data/FIG22-d-GNDL2new.png]

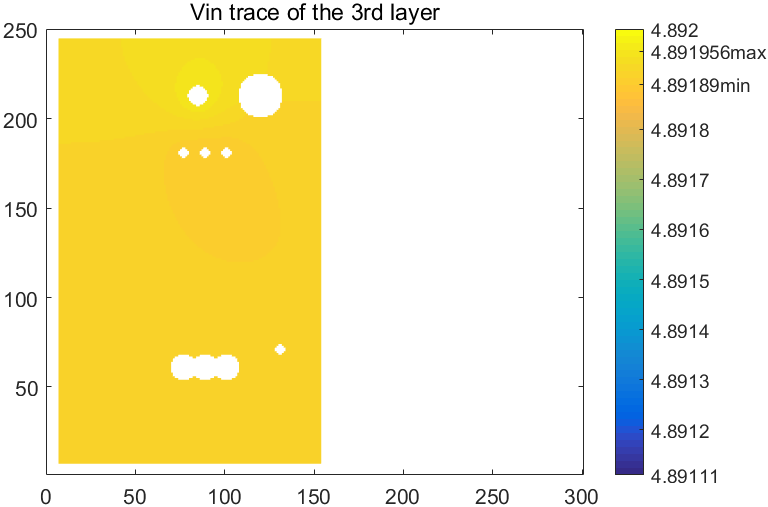

Supplement: S1 Data — (ZIP) [file pone.0310237.s001.zip › new figure data/FIG22-e-VinL3new.png]

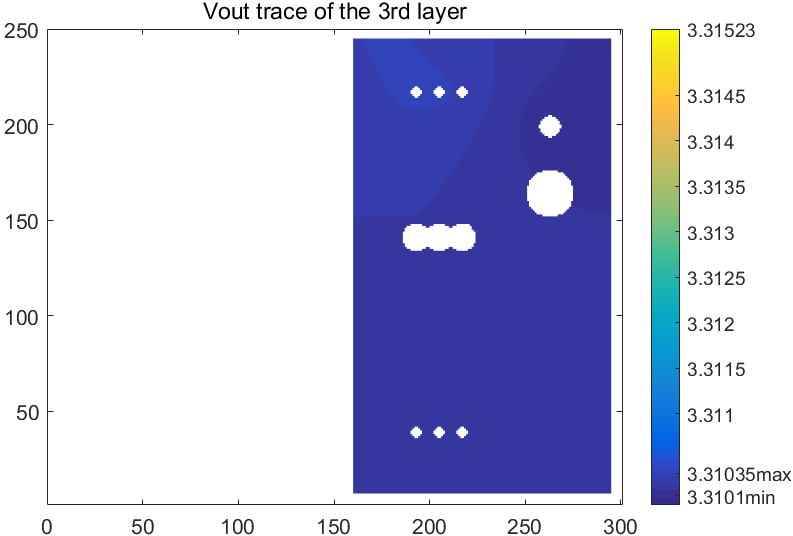

Supplement: S1 Data — (ZIP) [file pone.0310237.s001.zip › new figure data/FIG22-f-VoL3new.png]

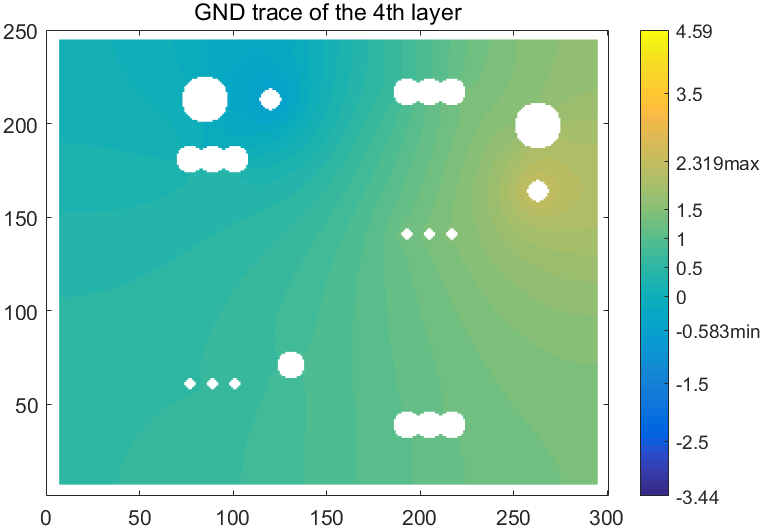

Supplement: S1 Data — (ZIP) [file pone.0310237.s001.zip › new figure data/FIG22-g-GNDL4new.png]

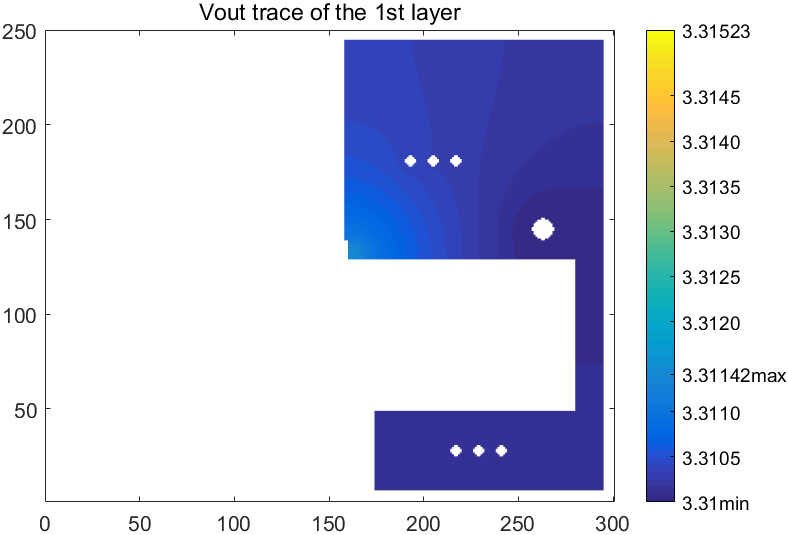

Supplement: S1 Data — (ZIP) [file pone.0310237.s001.zip › new figure data/FIG24-a-VoL1newLayout-nn.png]

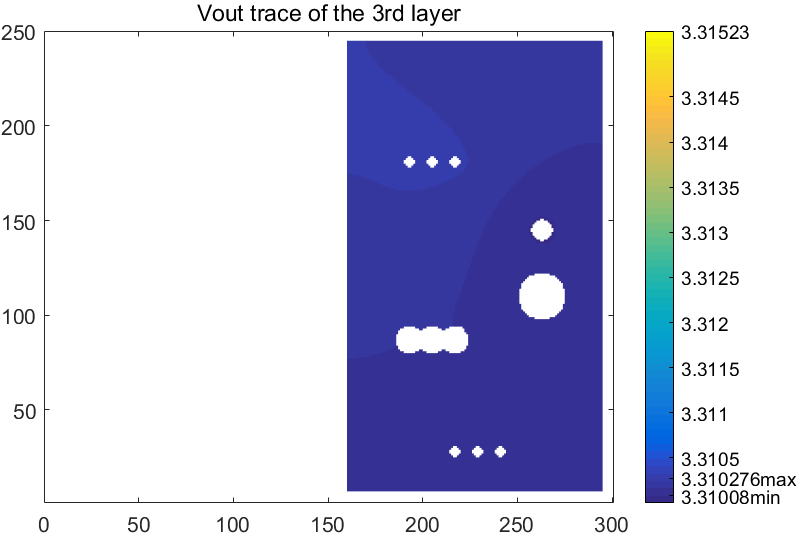

Supplement: S1 Data — (ZIP) [file pone.0310237.s001.zip › new figure data/FIG24-b-VoL3newLayout-nn.png]

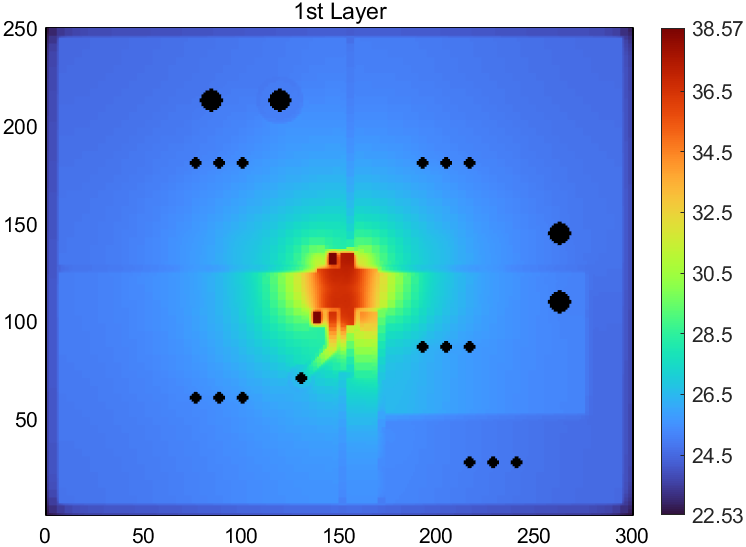

Supplement: S1 Data — (ZIP) [file pone.0310237.s001.zip › new figure data/FIG25-T1newLayout.png]
